# Adeno-associated-virus-mediated delivery of CRISPR-CasRx induces efficient RNA knockdown in the mouse testis

**DOI:** 10.7150/thno.95633

**Published:** 2024-06-17

**Authors:** Kai Li, Mengzhen Li, Yanyun Luo, Dingfeng Zou, Yahui Li, Xinyu Mang, Zexuan Zhang, Pengyu Li, Yan Lu, Shiying Miao, Wei Song

**Affiliations:** Department of Biochemistry and Molecular Biology, State Key Laboratory of Common Mechanism Research for Major Diseases, Institute of Basic Medical Sciences, Chinese Academy of Medical Sciences and Peking Union Medical College, Beijing 100005, China.

**Keywords:** AAV9-CRISPR-CasRx, RNA knockdown, 3-MA, germ-cell-specific transgene, spermatogenesis

## Abstract

**Rationale:** In male mammals, many developmental-stage-specific RNA transcripts (both coding and noncoding) are preferentially or exclusively expressed in the testis, where they play important roles in spermatogenesis and male fertility. However, a reliable platform for efficiently depleting various types of RNA transcripts to study their biological functions during spermatogenesis *in vivo* has not been developed.

**Methods:** We used an adeno-associated virus serotype nine (AAV9)-mediated CRISPR-CasRx system to knock down the expression of exogenous and endogenous RNA transcripts in the testis. Virus particles were injected into the seminiferous tubules via the efferent duct. Using an autophagy inhibitor, 3-methyladenine (3-MA), we optimized the AAV9 transduction efficiency in germ cells *in vivo*.

**Results:** AAV9-mediated delivery of CRISPR-CasRx effectively and specifically induces RNA transcripts (both coding and noncoding) knockdown in the testis *in vivo.* In addition, we showed that the co-microinjection of AAV9 and 3-MA into the seminiferous tubules enabled long-term transgene expression in the testis. Finally, we found that a promoter of *Sycp1* gene induced CRISPR-CasRx-mediated RNA transcript knockdown in a germ-cell-type-specific manner.

**Conclusion:** Our results demonstrate the efficacy and versatility of the AAV9-mediated CRISPR-CasRx system as a flexible knockdown platform for studying gene function during spermatogenesis *in vivo*. This approach may advance the development of RNA-targeting therapies for conditions affecting reproductive health.

## Introduction

The testis has a highly complex transcriptome, in terms of the number of coding or non-coding genes expressed 1. Single-cell RNA sequencing (scRNA-seq) of both human and murine germ cells has revealed that these cells exhibit distinct transcriptional signatures during spermatogenesis 2-6; however, whether these widespread transcriptional products play a functional role in spermatogenesis and fertilization requires further experimental investigation *in vivo*. Although the CRISPR/Cas9 system, a widely used technology for genome engineering, is a valuable tool for studying gene function *in vivo* during spermatogenesis, the permanent deletion of targeted genes may result in embryonic lethality or overt phenotypic changes; these factors may obscure genuine biological processes and features of gene expression regulation 7, 8. Additionally, the application of the CRISPR/Cas9 system for gene knockout studies of spermatogenesis is limited by certain genomic loci of non-coding genes, such as overlapping DNA regulatory elements 9, 10. As such, there is an unmet need for additional loss-of-function tools to interrogate gene function during spermatogenesis *in vivo*.

Local ''RNA knockdown'' using tools such as RNA interference (RNAi) or antisense oligonucleotides (ASOs) provides a highly complementary approach for depleting RNA, including long non-coding RNA (lncRNA), messenger RNA (mRNA), and circular RNA 11-13. In our previous reports, we established a strategy to identify functional lncRNAs during spermatogenesis *in vivo* using the adeno-associated virus (AAV)-mediated delivery of short-hairpin RNAs (shRNAs) 14. In addition, an ASO-based approach has been used to successfully knockdown lncRNA in the mouse testes 15. More recently, CRISPR-Cas13d, has been employed as a novel RNA-guided RNA-targeting system, to efficiently deplete RNA transcripts in mammalian cell lines and an animal model. Cas13d, a class 2 type VI single-effector CRISPR ribonuclease, can be directed to cleave single-stranded RNA via an engineered spacer within the guide RNA (gRNA), which is complementary to the sequence of its RNA targets. Among the known members of the Cas13d family, the Cas13d derived from *Ruminococcus flavefaciens* XPD3002 (CasRx), demonstrates superior RNA knockdown efficiency and specificity to those of RNAi 8, 16-21. Additionally, its small size (~966 amino acids) allows for convenient packaging into the AAV for transgene delivery *in vivo* 16. The AAV9-mediated CRISPR-CasRx system has recently been used to achieve the functional knockdown of RNA transcripts in a mouse model and, therefore, has therapeutic potential in the treatment of certain hereditary diseases 22-24. Given the preliminary success of this strategy, we sought to test whether AAV9-mediated CRISPR-CasRx could be delivered to the testis to induce endogenous and exogenous knockdown of RNA targets, and consequently, accelerate and simplify the process of gene function interrogation in this organ *in vivo*.

## Results

### Optimization of AAV9-mediated gene transduction of germ cells *in vivo*

**Figure 1:**
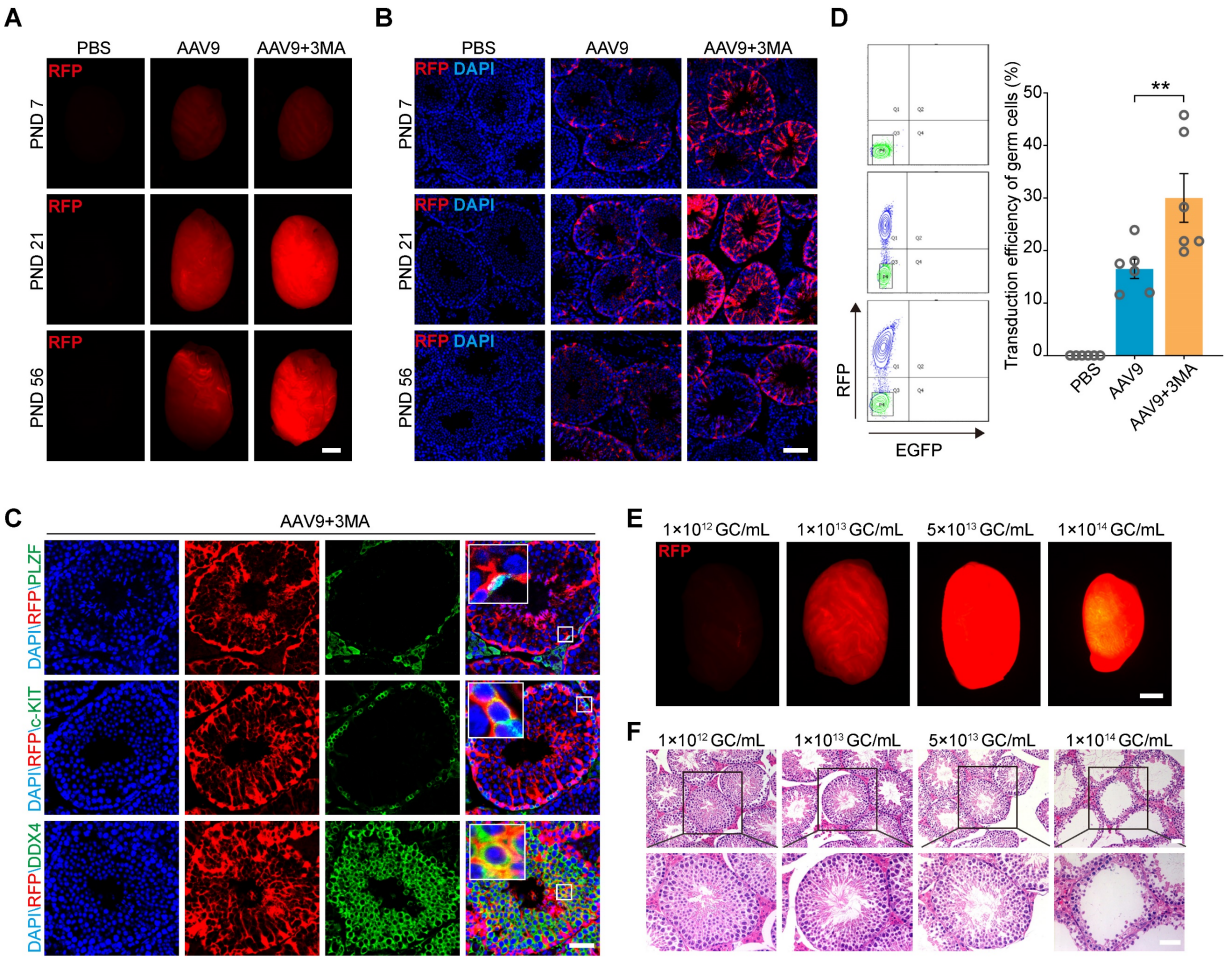
** Co-injection of AAV9 and 3-MA increase the transduction efficiency of spermatogenic cells *in vivo*. (A)** Stereomicroscopic fluorescence imaging of representative testes (postnatal days [PND] 7, 21, and 56) 4 weeks after the microinjection of PBS, AAV9-CMV-RFP, or AAV9-CMV-RFP+3-MA. RFP: red fluorescence protein reporter gene. Scale bar: 1 mm. **(B)** Immunostaining of testicular sections for RFP (red) derived from testes (PND 7, 21, and 56) microinjected with PBS, AAV9-CMV-RFP, or AAV9-CMV-RFP+3-MA. Nuclei were stained with DAPI (blue). Scale bar: 100 μm. **(C)** Immunostaining of testicular sections from testes co-injected with AAV9-CMV-RFP and 3-MA for RFP (red), c-KIT (green, top), and DDX4 (green, bottom). Nuclei were stained with DAPI (blue). Scale bar: 50 μm. **(D)** Flow cytometric analysis of the percentage of RFP^+^ germ cells in testes 4 weeks after microinjection of PBS, AAV9-CMV-RFP, or AAV9-CMV-RFP+3-MA. *Left*: representative flow cytometry contour plot; *Right*: quantification of the percentage of RFP^+^ germ cells; Data are presented as the mean ± SEM (*n* = 6). *P*-values were determined using the two-tailed Student's *t*-test; **P < 0.01. **(E)** Stereomicroscopic fluorescence imaging of representative testes co-injected with AAV9-CMV-RFP and 3-MA at the indicated AAV titer. Scale bar: 1 mm. **(F)** H&E staining of testicular sections from testes co-injected with AAV9-CMV-RFP and 3-MA at the indicated AAV titer. Scale bar: 50 μm.

The tubular injection of AAV9 transduces both germ and Sertoli cells; however, the transduction efficiency of germ cells remains suboptimal 25. Inhibiting autophagy can increase the efficiency of exogenous DNA electroporation or mumps virus (MuV) infection into male germ cells 26, 27. Thus, to optimize AAV9-mediated delivery of CRISPR-CasRx to the testis *in vivo* and subsequently improve the AAV9 transduction efficiency of this organ, we co-injected AAV9-CMV-RFP with 3-methyladenine (3-MA), a widely used autophagy inhibitor, into the seminiferous tubules of testis on postnatal days (PND) 7, 21, and 56. We then checked the fluorescence intensity of the red florescence protein (RFP) reporter by both fluorescence stereomicroscopy and immunostaining 4 weeks after each microinjection. The results showed that the co-injection of AAV9-CMV-RFP and 3-MA substantially increased RFP expression in the testis from PND 21 onward (Figure 1A-B). In order to identify the expression characteristics of AAV9-CMV-RFP during mouse spermatogenesis, we tested its expression in spermatogenic cells by immunofluorescence staining. We observed the co-localization of the RFP signal with that of PLZF (a marker for undifferentiated spermatogonia), c-KIT (a marker for differentiating spermatogonia) and DDX4 (a marker for spermatogenic cell), suggesting that AAV9-mediated gene transfer had occurred in the germ cells (Figure 1C). To quantify the transduction efficiency of co-injecting AAV9 and 3-MA into spermatogenic cells, we depleted the somatic cells and quantified the percentage of RFP^+^ germ cells using flow cytometry. The results showed that the transduction efficiency of AAV9+3-MA in spermatogenic cells was significantly higher than that of AAV9 alone (AAV9+3-MA, mean ~30%; AAV9 alone, mean ~16.5%; *P* < 0.01) (Figure 1D). Because the AAV9 vector can be administered at relatively high doses, we next evaluated how testis transduction efficiency varied at different AAV9 titers. Stereomicroscopic fluorescence imaging showed that a titer of 1-5 × 10^13^ genome copies (GC)/mL resulted in high RFP expression; moreover, the hematoxylin and eosin (H&E) staining of testicular sections showed that spermatogenesis was not significantly affected at this AAV9 dose (Figure 1E-F). Although a titer of 1 × 10^14^ GC/mL elicited the strongest RFP fluorescence, it induced marked developmental defects in spermatogenesis, such as testicular shrinkage and germ cell loss. In view of this, we selected a titer of 1 × 10^13^ GC/mL AAV9 for subsequent transgene delivery experiments. Our findings suggest that 3-MA improves AAV9 transduction efficiency in germ cells *in vivo* and that AAV9 is safe to use within the specified titer range.

### *In vivo* kinetics and safety assessment of AAV9 and 3-MA co-injection into the testis

**Figure 2:**
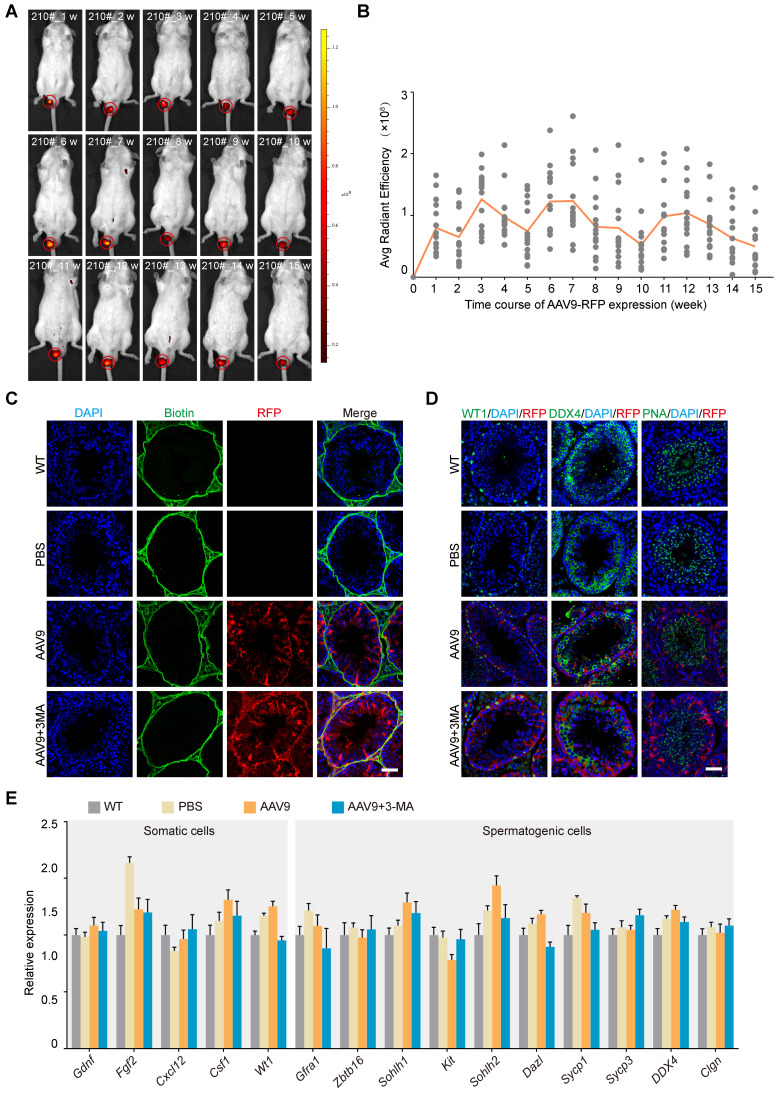
***In vivo* kinetics and safety assessment of the co-injection of AAV9 and 3-MA into the testis. (A)** Representative sequential fluorescence imaging of the RFP signal (in mouse 210#) at indicated time-points (weeks 1-15) after co-injection of AAV9-CMV-RFP+3-MA, determined using an *in vivo* imaging system (IVIS). Dark red and yellow indicate low and high *RFP* expression, respectively, following AAV9-CMV-RFP administration. **(B)** Kinetics of AAV9-CMV-RFP expression in the mouse testis *in vivo* at the indicated time-points. Average (Avg) radiant efficiency was determined from the fluorescence imaging results. Sequential fluorescence imaging data were generated from each mouse at each time-point (*n* = 15). **(C)** Results of the biotin tracer experiment to determine BTB integrity. The mouse testes (at PND 21) were first microinjected with PBS, AAV9-CMV-RFP, or AAV9-CMV-RFP+3-MA and then interstitially injected with biotin (green). Samples were recovered 30 min after biotin microinjection (*n* = 3). Scale bar: 50 μm. **(D)** Immunostaining of testicular sections for WT1 (green, left), DDX4 (green, middle), and PNA (green, right) from testes microinjected with PBS, AAV9-CMV-RFP, or AAV9-CMV-RFP+3-MA. Nuclei were stained with DAPI (blue). Scale bar: 50 μm. **(E)** qRT-PCR analysis of representative functional gene expression in somatic and spermatogenic cells isolated from testes microinjected with PBS, AAV9-CMV-RFP, or AAV9-CMV-RFP+3-MA. Data were normalized to *Actb* expression and are presented as the mean ± SEM (*n* = 3).

Our next objective was to characterize the AAV9 transduction kinetics in the mouse testis. To this end, we investigated the tissue expression profile of AAV9-CMV-RFP using stereomicroscopy and western blotting after microinjecting AAV9 and 3-MA into the seminiferous tubules of the testis. The results showed that RFP expression was localized to the mouse testis and negligible in other organs (Figure S1A-B). We then tested whether AAV9-mediated transgene expression persisted long-term by tracking the dynamics of the RFP signal in the mouse testis over time. Continuous *in vivo* bioluminescence imaging and a semi-quantitative analysis of radiant efficiency revealed that RFP expression was detected 1 week after microinjection, peaked after 3-4 weeks, and then gradually decreased until week 5. Notably, we observed that RFP expression assumed a rhythmic profile in the mouse testis, whereby its expression rose again on week 6, before gradually declining in week 7. Interestingly, this 5-week cycle aligned with the spermatogenic wave pattern of mice and was maintained for at least 15 weeks, suggesting that AAV9 induced long-term transgene expression in the testis (Figure 2A-B). AAV9 penetrates the tight junctions of the blood-testis barrier (BTB) without perturbing their structure; in addition, it exerts no apparent toxicity on somatic and spermatogenic cells. We therefore next assessed whether AAV9 co-injection with 3-MA impaired the integrity of the BTB and induced potential toxic effects on germline developmental progression *in vivo*. To this end, we performed a biotin tracer experiment in the testis to test the integrity of the BTB and immunostaining for DDX4, PNA (a post-meiotic germ cell marker) and WT1 (a Sertoli cell marker) 3 weeks after AAV9 and 3-MA co-injection. The biotin tracer experiment revealed that the biotin was localized to the interstitial area and the basement membrane, and did not enter the lumen of the seminiferous tubules. This finding suggested that the co-injection of AAV9 and 3-MA had no effect on the integrity of the BTB (Figure 2C). In addition, the expression of DDX4, PNA, and WT1 was not significantly different between the AAV9+3-MA and AAV9 alone groups, indicating that the combination treatment did not hamper germline developmental progression *in vivo* (Figure 2D). Next, we examined the number and morphological development of mature spermatozoa. The results showed that there were no significant differences in the number of mature spermatozoa among WT group, AAV9 group and AAV9+3MA group (Figure S1C). Immunostaining for PNA showed the normal morphology of mature spermatozoa from testis treated with AAV9+3MA (Figure S1D). We then examined the expression levels of genes encoding several representative cytokines from the testicular microenvironment (e.g., *Gdnf*,* Fgf2*, *Cxcl12*, *Csf1*), as well as that of functional spermatogenic genes (e.g., *Zbtb16*, *Sohlh1*, *Kit*, *Dazl*, *Sycp1*, *Sycp3*, *Ddx4*, *Clgn*), using quantitative real-time PCR (qRT-PCR). We found that the expression levels of these genes were largely unaltered following AAV9 and 3-MA co-injection (Figure 2E), suggesting that this combination had little effect on the testicular microenvironment and endogenous gene expression in spermatogenic cells. Taken together, these findings demonstrate that AAV9 and 3-MA co-injection provided long-term transgene expression without adversely affecting germline development *in vivo*.

### AAV9-mediated CRISPR-CasRx induces reporter mRNA knockdown in the testis *in vivo*

**Figure 3:**
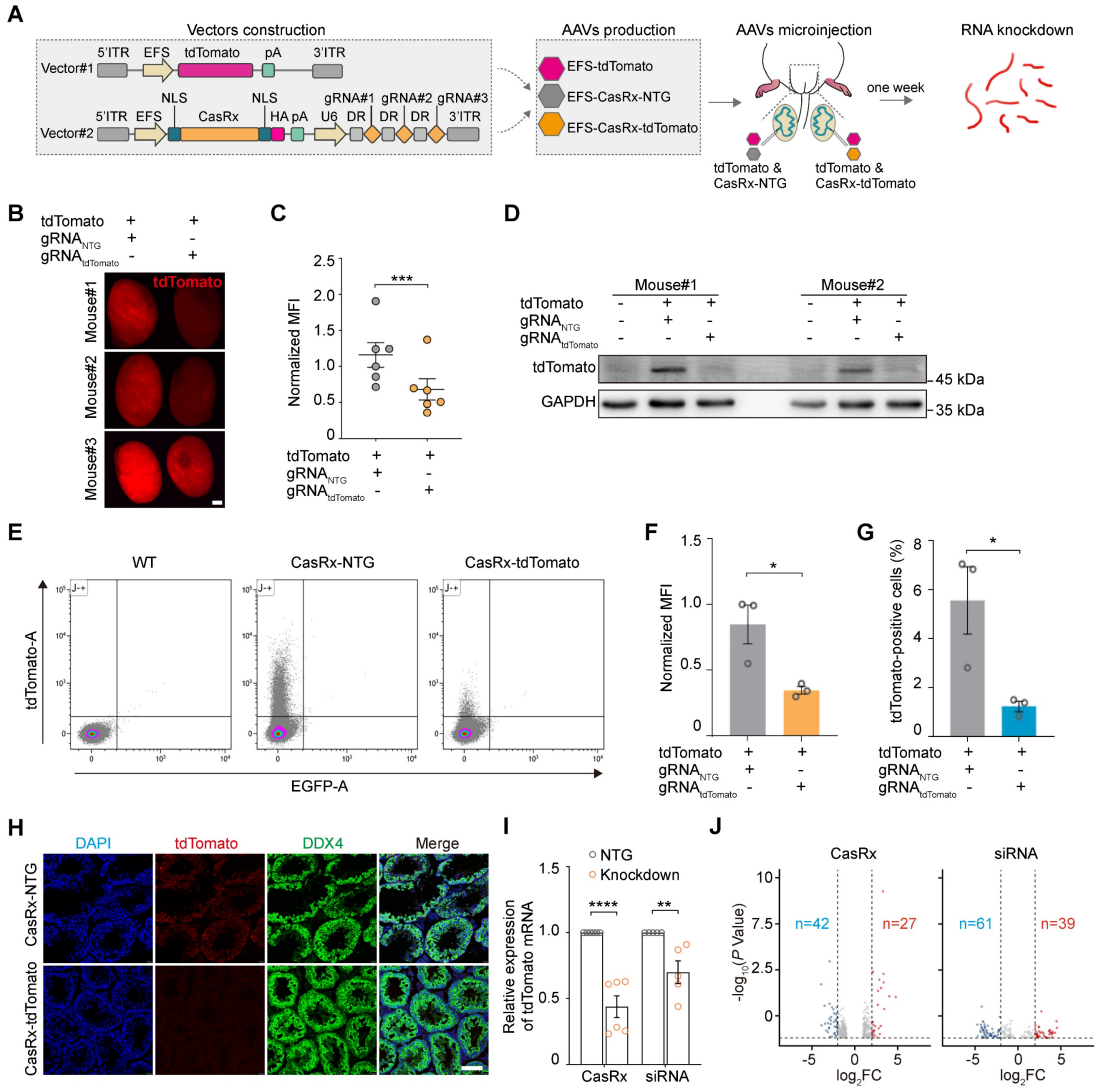
** AAV9-mediated CRISPR-CasRx specifically targets reporter mRNA expression in the mouse testis *in vivo*. (A)** Schematic illustration of reporter mRNA knockdown by AAV9-mediated CRISPR-CasRx *in vivo*. Vector#1 (AAV9-EFS-tdTomato) encodes the tdTomato reporter, driven by the EFS promoter; Vector#2 (AAV9-EFS-CasRx-tdTomato) encodes CasRx and gRNAs. **(B)** Stereomicroscopic fluorescence imaging of representative testes microinjected with AAV9-EFS-CasRx-tdTomato or AAV9-EFS-CasRx-NTG together with AAV9-EFS-tdTomato. Scale bar: 1 mm. **(C)** Mean fluorescence intensity (MFI) of tdTomato in testes microinjected with either AAV9-EFS-CasRx-tdTomato or AAV9-EFS-CasRx-NTG together with AAV9-EFS-tdTomato. Data were normalized to the tdTomato-non-targeting condition (*n* = 6). **(D)** Western blotting analysis of tdTomato protein expression following AAV9-EFS-CasRx-tdTomato treatment. GAPDH was served as the loading control. Data from two biologically independent samples are shown. **(E)** Representative flow cytometry dot plot showing the percentage of tdTomato-positive cells in the testes microinjected with AAV9-EFS-CasRx-tdTomato or AAV9-EFS-CasRx-NTG together with AAV9-EFS-tdTomato. **(F and G)** Flow cytometric analysis of the tdTomato MFI and the percentage of tdTomato^+^ cells from the testes microinjected with AAV9-EFS-CasRx-tdTomato or AAV9-EFS-CasRx-NTG together with AAV9-EFS-tdTomato. Data were normalized to the tdTomato-non-targeting condition (*n* = 3). **(H)** Immunostaining of testicular sections for tdTomato (red) and DDX4 (green). Nuclei were stained with DAPI (blue). Scale bar: 100 μm. **(I)** qRT-PCR analysis of tdTomato mRNA knockdown efficiency by individual position-matched gRNAs and siRNAs. Data were normalized to *Gapdh* expression (n >3). **(J)** Volcano plots of differential transcript levels between tdTomato-targeting and NTG CasRx (left) or tdTomato-targeting and non-targeting siRNA (right) as determined by RNA sequencing. All data are presented as the mean ± SEM. *P*-values were determined using the two-tailed Student's *t*-test; *P < 0.05; **P < 0.01; ***P < 0.001; ****P < 0.0001.

CRISPR-CasRx offers an effective approach for knocking down specific RNA transcripts in individual mammalian cells (e.g., the neuron) or organs (e.g., the liver), as well as on a systemic level (i.e., in the embryo) 8, 16, 19. Meanwhile, AAV is a promising vehicle for *in vivo* transgene delivery and gene therapy 16. Thus, we next determined the RNA-targeting capabilities of the AAV9-mediated delivery of CRISPR-CasRx into the mouse testis. To achieve this, we first constructed an AAV expression vector encoding the tdTomato fluorescence reporter (AAV9-EFS-tdTomato). Next, we designed a set of three 30-nucleotide gRNAs targeting the coding sequence of tdTomato mRNA; an expression vector carrying tdTomato-non-targeting gRNAs (AAV9-EFS-CasRx-NTG) was used as a negative control. We then constructed the all-in-one AAV expression vector which contained a gRNA array as well as an mRNA encoding the CasRx protein (AAV9-EFS-CasRx-tdTomato) (Figure 3A). To examine the efficacy of the gRNAs at knocking down the expression of tdTomato, we co-transfected human embryonic kidney (HEK) 293T cells with AAV9-EFS-tdTomato and AAV9-EFS-CasRx-tdTomato or AAV9-EFS-CasRx-NTG. Fluorescence imaging and western blotting showed that the expression of tdTomato decreased dramatically following the transfection of tdTomato-targeting gRNA but not after the transfection of the NTG (Figure S2A-B). The above vectors were then packaged into AAV9 and used in bilateral testicular microinjection experiments, whereby one testis of a 3-week-old mouse was co-injected (into the seminiferous tubule) with AAV9-EFS-tdTomato and AAV9-EFS-CasRx-NTG, while the contralateral testis received AAV9-EFS-tdTomato and AAV9-EFS-CasRx-tdTomato. The CasRx-mediated knockdown efficiency of tdTomato was then examined 1 week after the co-injection. Fluorescence stereomicroscopy showed that the mean fluorescence intensity (MFI) of the testis co-injected with AAV9-EFS-tdTomato and AAV9-EFS-CasRx-tdTomato was ~50% lower than that of the control testis (Figure 3B-C); Western blotting further confirmed that CRISPR-CasRx caused a 2-fold reduction in tdTomato protein expression in the testis (Figure 3D). These findings demonstrate that AAV9-mediated CRISPR-CasRx effectively lowers tdTomato protein level in the testis *in vivo*.

We next used flow cytometry to quantify tdTomato fluorescence intensity and further evaluate the knockdown efficiency and specificity of CRISPR-CasRx in the testis *in vivo* (Figure 3E). We found that CRISPR-CasRx significantly altered the level of tdTomato expression in testicular cells. Notably, the administration of AAV9-EFS-CasRx-tdTomato reduced tdTomato fluorescence intensity by ~50% (Figure 3F), while the percentage of tdTomato^+^ testicular cells was decreased by ~75% (Figure 3G). Additionally, immunostaining showed that there was no substantial difference in DDX4 expression between the testis of the AAV9-EFS-CasRx-NTG- and AAV9-EFS-CasRx-tdTomato-treated groups of mice, other than the marked reduction in tdTomato expression (Figure 3H). These findings confirm that AAV9-mediated CRISPR-CasRx achieved effective knockdown of tdTomato expression in the testis *in vivo*.

To further compare the efficiency and specificity of RNA knockdown achieved by CRISPR-CasRx and siRNA, we designed position-matched gRNA and siRNA to target reporter tdTomato mRNA (Figure S2C-E). We observed that compared to siRNA, CRISPR-CasRx showed higher efficiency of RNA knockdown (CRISPR-CasRx, mean ~60%; siRNA, mean ~30%) in the testis *in vivo* (Figure 3I). We next performed RNA-seq to test the possible off-target effects of CRISPR-CasRx and siRNA. Differential expression analysis indicated that CRISPR-CasRx exerts fewer off-target effects than that of siRNA in the testis *in vivo* (Figure 3J). Importantly, tdTomato was the most significantly downregulated transcript (foldchange > 5), whereas the transcriptional levels of typical functional spermatogenic genes remained largely unchanged in CRISPR-CasRx condition (Figure S2F-G). Collectively, these results suggest that AAV9-mediated CRISPR-CasRx is a highly effective and safe RNA knockdown tool for use in the testis *in vivo*.

### AAV9-mediated CRISPR-CasRx effectively silences endogenous functional spermatogenic genes in the testis *in vivo*

**Figure 4:**
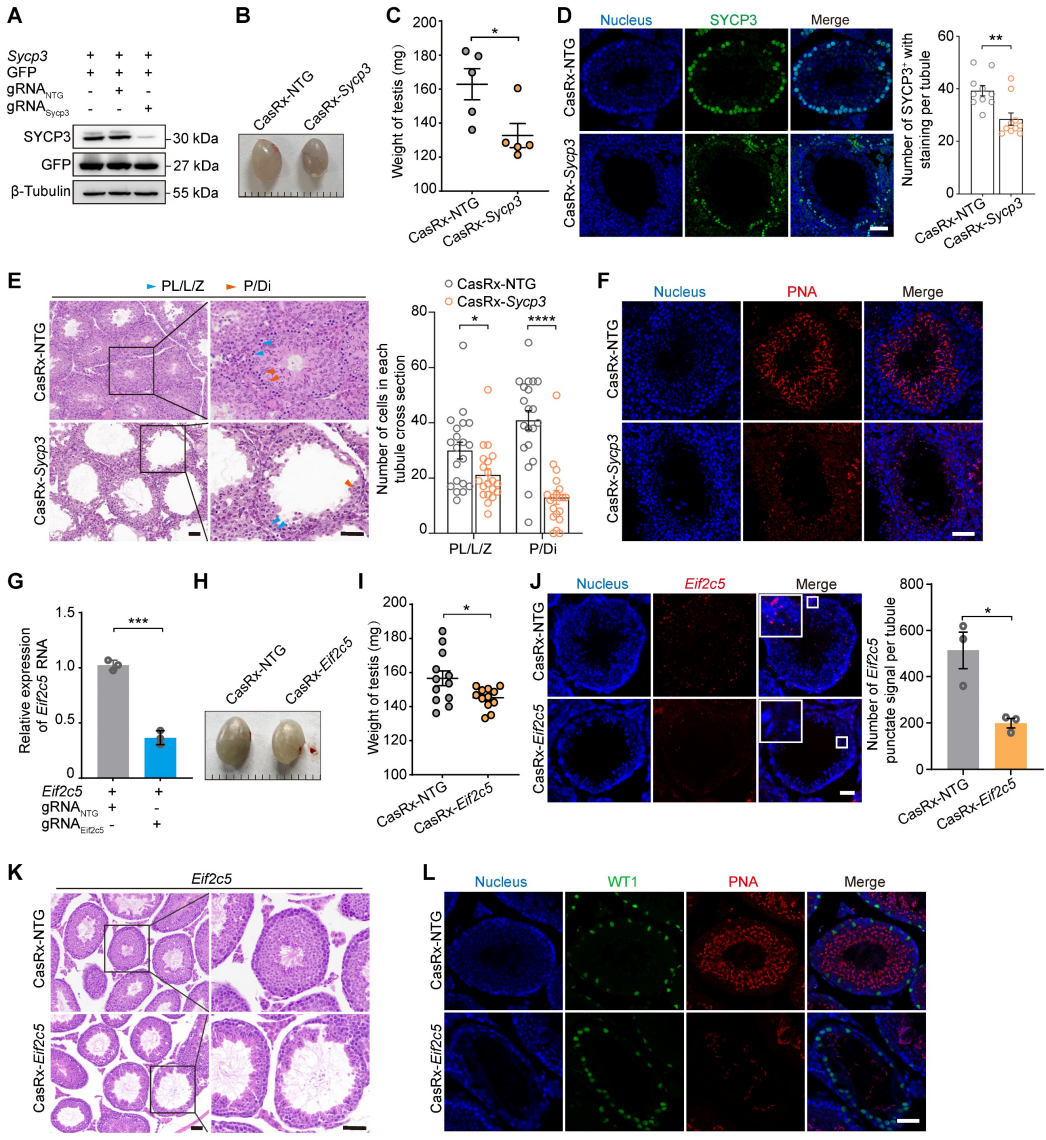
** Transcriptome editing of Sycp3 and lncRNA-Eif2c5 by AAV9-mediated CRISPR-CasRx in the testis* in vivo*. (A)** Western blotting analysis of SYCP3 protein expression in HEK293T cells after CRISPR-CasRx gene editing. GFP was used as transfection control. β-Tubulin was used as a loading control. **(B and C)** Comparison of the size (B) and weight (C) of testes from control (CasRx-NTG) and SYCP3-depleted (CasRx-*Sycp3*) mice (*n* = 5). Scale bar: 1 mm. **(D)** Immunostaining of testicular sections from the control (CasRx-NTG) and SYCP3-depleted (CasRx-*Sycp3*) mice for SYCP3 (green). Nuclei were stained with DAPI (blue). Scale bar: 50 μm. *Left:* representative images; *Right:* number of stained cells per tubule (*n* = 10). **(E)** H&E staining of testicular sections from the control (CasRx-NTG) and SYCP3-depleted (CasRx-*Sycp3*) mice. Scale bar: 50 μm. *Left:* representative images; *Right:* number of Sub-stages of the prophase I spermatocytes (pre-leptotene, leptotene and zygotene spermatocytes [PL/L/Z]; pachytene and diplotene spermatocytes [P/DI]) with staining per tubule (*n* = 10). **(F)** Immunostaining of testicular sections from the control (CasRx-NTG) and SYCP3-depleted (CasRx-*Sycp3*) mice for PNA (red). Nuclei were stained with DAPI (blue). Scale bar: 50 μm. **(G)** qRT-PCR analysis of lncRNA-*Eif2c5* knockdown efficiency in HEK293T cells transfected with vectors expressing *Eif2c5*, CasRx, and gRNAs. Data were normalized to* Actb* expression (*n* = 3). **(H and I)** The size (H) and weight (I) of testes from the control (CasRx-NTG) and *Eif2c5*-depleted (CasRx-*Eif2c5*) mice (*n* =12). **(J)** RNAscope analysis of lncRNA-*Eif2c5* (red) targeted by AAV9-mediated CRISPR-CasRx *in vivo*. Nuclei were stained with DAPI (blue). Scale bar: 50 μm. *Left*: representative images; *Right*: quantification of *Eif2c5* punctate signal per tubule. **(K)** H&E staining of testicular sections from the control (CasRx-NTG) and *Eif2c5*-depleted (CasRx-*Eif2c5*) mice. Scale bar: 50 μm. **(L)** Immunostaining of testicular sections from the control (CasRx-NTG) and *Eif2c5*-depleted (CasRx-*Eif2c5*) mice for WT1 (green) and PNA (red). Nuclei were stained with DAPI (blue). Scale bar: 50 μm. All data are presented as the mean ± SEM. *P*-values were determined using the two-tailed Student's *t*-test; *P < 0.05; **P < 0.01; ***P < 0.001.

To better understand the roles of specific genes during spermatogenesis, we next evaluated the ability of the AAV9-mediated CRISPR-CasRx system to silence endogenous spermatogenic genes. Our previous work showed that the shRNA-mediated knockdown of *Sycp3* mRNA led to spermatogenic defects 14; thus, we decided to target *Sycp3* mRNA to investigate its potential application within the CRISPR-CasRx system in testis *in vivo*. To identify gRNAs targeting *Sycp3* mRNA, we transfected HEK293T cells with an expression vector encoding *Sycp3* (EFS-*Sycp3*), as well as an all-in-one vector expressing CasRx and gRNAs (AAV9-EFS-CasRx-*Sycp3* or AAV9-EFS-CasRx-NTG). qRT-PCR and western blotting results showed a significant decrease (~90%) in *Sycp3* expression 2 days after transfection (Figure 4A; Figure S3A). We then packaged the above vectors in AAV9 and microinjected them into each testis of a 3-week-old mouse. We found that, 3 weeks after the microinjection, the testis treated with AAV9-EFS-CasRx-*Sycp3* was smaller and weighed significantly less (~25%) than that treated with the CasRx-NTG control (Figure 4B-C). Immunofluorescence staining for SYCP3 revealed a significant decrease in the number of SYCP3^+^ cells and the corresponding fluorescence intensity of the CasRx-Sycp3-treated testes, suggesting that CasRx successfully knocked down *Sycp3* in the testis *in vivo* (Figure 4D). H&E staining of testicular sections revealed that the CasRx-*Sycp3*-treated testes exhibited histological defects, characterized by a thinner seminiferous epithelium and germ cell loss, compared to the control testes (Figure 4E). To further characterize these spermatogenic defects, we quantified the number of the spermatocyte populations (pre-leptotene, leptotene and zygotene spermatocytes [PL/L/Z]; pachytene and diplotene spermatocytes [P/Di]) based on morphological analysis of testicular sections 28. The result showed a significant decrease in the number of pachytene and diplotene spermatocytes (P/Di) (Figure 4E). Additionally, we performed immunofluorescence staining for PNA and WT1 of testicular sections from CasRx-*Sycp3*- and CasRx-NTG-treated mice. Although we found no significant difference in the number of Sertoli cells between these two groups of mice, the number of post-meiotic germ cells was substantially reduced in the testes of CasRx-*Sycp3*-treated mice, indicating spermatogenesis dysfunction in these animals (Figure 4F; Figure S3B).

RNAi-based methods (siRNA or shRNA) are often more effective against RNA localized to the cytoplasm than that localized to the nucleus 29. Therefore, we next sought to determine whether CRISPR-CasRx could achieve specific and efficient knockdown of nuclear RNA in the testis *in vivo*. To this end, we targeted a nuclear lncRNA of metastasis-associated lung adenocarcinoma transcript 1 (*Malat1*), a gene previously reported to have no impact on reproductive development 30. We used qRT-PCR to show that CRISPR-CasRx decreased lncRNA-*Malat1* levels by ~70% in GC1-spg cells and by ~50% in the testis *in vivo*, indicating that this approach achieved successful knockdown of an RNA localized to the nucleus (Figure S3C-D). Our previous work had revealed the important regulatory role of lncRNA-*Eif2c5* in mouse spermatogenesis 12. We therefore designed gRNAs targeting lncRNA-*Eif2c5* and examined how well CRISPR-CasRx knocked down its expression using qRT-PCR; we observed a ~60% decrease in HEK293T cells and a ~40% decrease in the testis *in vivo* (Figure 4G; Figure S3E). Additionally, we found that CasRx-mediated *Eif2c5* knockdown (CasRx-*Eif2c5*) reduced testis weight by ~25% relative to that of the CasRx-NTG control (Figure 4H-I). We next performed RNAscope *in situ* hybridization to evaluate the knockdown efficiency of *Eif2c5* and found that CRISPR-CasRx visibly decreased the *Eif2c5* punctate signal (~60%) (Figure 4J). H&E staining of testicular sections revealed that the CasRx-*Eif2c5*-treated testes exhibited severe morphological defects and had markedly fewer germ cells in their seminiferous tubules than the control testes (Figure 4K). Immunofluorescence staining of testicular sections from mice injected with CasRx-*Eif2c5* for PNA showed a significant reduction in the number of post-meiotic germ cells, indicative of disrupted spermatogenesis (Figure 4L). Collectively, these results demonstrate that the CRISPR-CasRx system can be delivered to the testis to effectively knock down endogenous spermatogenic genes, mRNA, and lncRNA, and therefore be used as a tool for studying gene function during spermatogenesis *in vivo*.

### AAV-mediated expression of the *Sycp1* promoter drives transgene expression in meiotic germ cells* in vivo*

**Figure 5:**
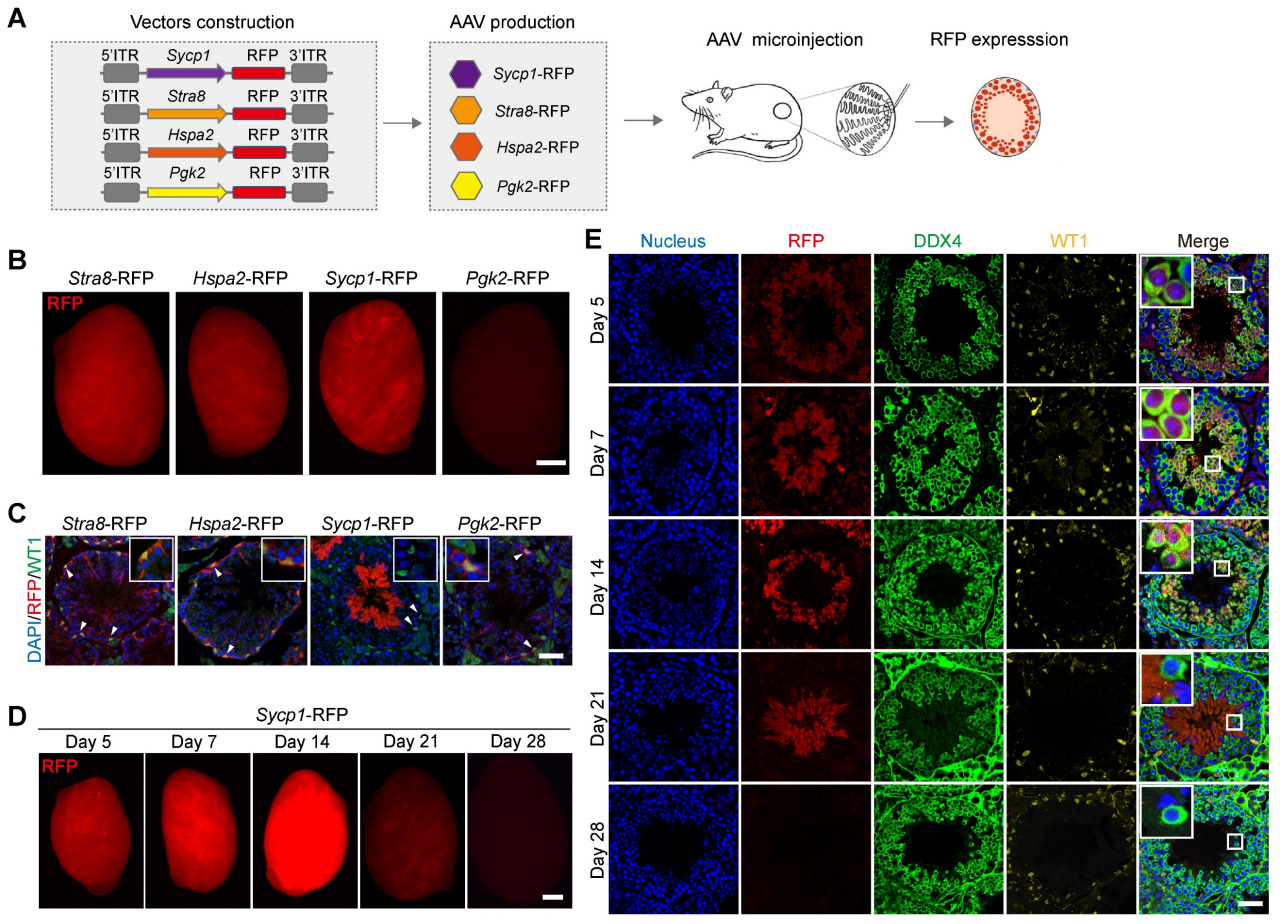
** AAV9 induces the Sycp1 promoter to drive transgene expression in germ cells *in vi*vo. (A)** Schematic illustration of the process used to screen for germ-cell-specific gene promoters that drive *RFP* reporter gene expression in distinct germ cell types *in vivo*; four independent germ-cell-specific gene promoter (i.e., *Stra8*, *Hspa2*, *Sycp1,* and *Pgk2*) were individually fused to the *RFP* reporter gene, packaged into AAV9, and then microinjected into the seminiferous tubule of testis of 3-week-old mice. **(B)** Stereomicroscopic fluorescence imaging of representative testes microinjected with AAV9-*Stra8*-RFP, AAV9-*Hspa2*-RFP, AAV9-*Sycp1*-RFP, or AAV9-*Pgk2*-RFP. Scale bar: 1 mm. **(C)** Immunostaining of testicular sections from testes received microinjection with AAV9-*Stra8*-RFP、AAV9-*Hspa2*-RFP、AAV9-*Sycp1*-RFP and AAV9-*Pgk2*-RFP for WT1 (green) and RFP (red). Nuclei were stained with DAPI (blue). Scale bar: 50 μm. **(D)** Representative stereomicroscopic fluorescent images of testes on days 5, 7, 14, 21, and 28 after microinjection of AAV9-*Sycp1*-RFP. Scale bar: 1 mm. **(E)** Immunostaining of testicular sections on days 5, 7, 14, 21, and 28 after microinjection of AAV9-*Sycp1*-RFP for WT1 (yellow), DDX4 (green), and RFP (red). Nuclei were stained with DAPI (blue). Scale bar: 50 μm.

The targeted delivery of genes to specific cell types is a critical aspect of AAV9-mediated gene therapy and regulation 31. Our next objective was therefore to screen germ-cell-specific gene promoters, which drive transgene expression, in distinct germ cell types *in vivo*. To this end, we generated four independent AAV expression vectors carrying germ-cell-specific gene promoter fragment fused to an RFP-reporter-encoding gene to generate AAV9-*Stra8*-RFP, AAV9-*Hspa2*-RFP, AAV9-*Sycp1*-RFP, and AAV9-*Pgk2*-RFP 32-35. These vectors were then packaged into AAV9 and used in testicular microinjection experiments, which measured the pattern of RFP expression in distinct germ cell types (Figure 5A). A strong RFP fluorescence signal was detected by fluorescence stereomicroscopy in the testis treated with AAV9-*Stra8*-RFP, AAV9-*Hspa2*-RFP, and AAV9-*Sycp1*-RFP at 3 weeks after microinjection; meanwhile, only weak RFP fluorescence intensity was observed in the testes treated with AAV9-*Pgk2*-RFP (Figure 5B). To further determine whether these promoters could drive transgene expression specifically in germ cells, we examined the distribution of RFP fluorescence in testicular sections subjected to immunofluorescence staining for RFP and WT1. Notably, we observed that RFP expression driven by AAV9-*Sycp1*-RFP was germ cell-specific, mainly in elongated spermatids and sperm at 3 weeks after microinjection. In contrast, AAV9-*Stra8*-RFP, AAV9-*Hspa2*-RFP and AAV9-*Pgk2*-RFP unexpectedly induced RFP expression in both germ and Sertoli cells (Figure 5C). Next, we characterized the temporal and spatial expression pattern of RFP fluorescence, driven by AAV9-*Sycp1*-RFP, during germ cell development *in vivo*. The fluorescence stereomicroscopy results showed a gradual increase in RFP fluorescence intensity in the testis on day 7 after microinjection of AAV9-*Sycp1*-RFP, which peaked on day 14 and gradually decrease until day 21 (Figure 5D). Immunofluorescence staining for DDX4 and WT1 showed that AAV9-*Sycp1*-RFP initiated RFP expression from day 5 to day 21 after microinjection, which persisted in spermatocytes, round spermatids, and elongating spermatids (Figure 5E).

We next wondered whether the AAV9-mediated germ-cell-specific transgene expression was driven by the *Sycp1* promoter; Thus, we generated an AAV expression vector carrying the *Sycp1* promoter fragment fused to a Cre recombinase gene (AAV9-*Sycp1*-Cre), packaged it into AAV9, and microinjecting it into the testis of 3-week-old Ai9 mice (Rosa26-CAG-LSL-tdTomato); we then characterized the temporal and spatial expression pattern of tdTomato fluorescence in the testis (Figure S4A). Using fluorescence stereomicroscopy, we observed that tdTomato fluorescence intensity gradually increased at day 7 post-microinjection, peaked on day 21, and significantly decreased by day 28 (Figure S4B). Next, we performed immunofluorescence staining of testicular sections for DDX4 and WT1 to determine the distribution of tdTomato fluorescence in distinct germ cell types. The tdTomato fluorescence was continuously detected from day 7 to day 21 after microinjection and persisted in spermatocytes, round spermatids, and elongating spermatids (Figure S4C). Taken together, these results demonstrate that the AAV9-mediated delivery of the germ-cell-specific *Sycp1* promoter could successfully drive transgene expression specifically in germ cells.

### The germ-cell-specific *Sycp1* promoter controls CRISPR-CasRx efficiently and induces reporter mRNA knockdown in germ cells *in vivo*

**Figure 6:**
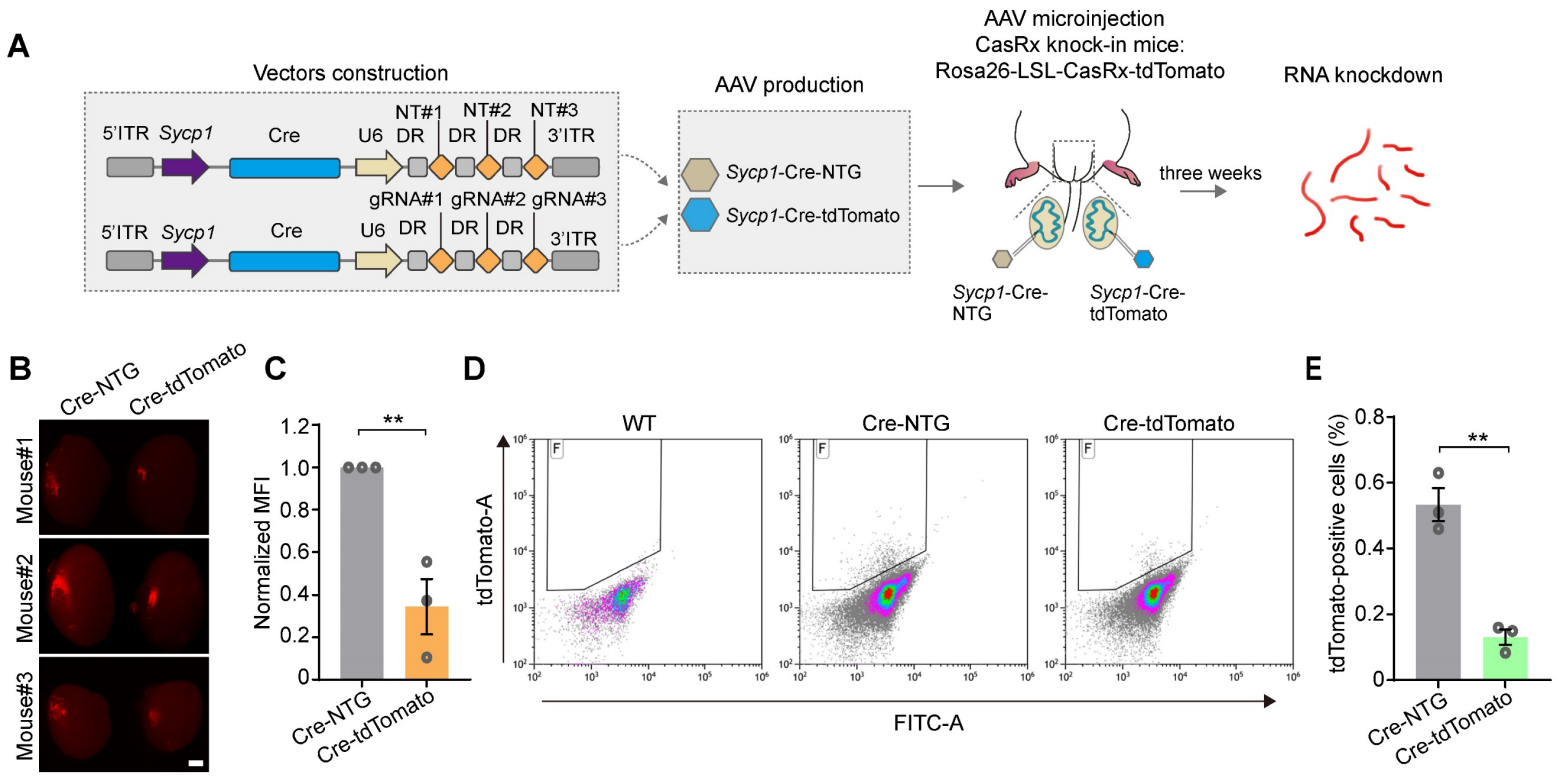
** The AAV9- and Sycp1-mediated induction of the CRISPR-CasRx system achieves RNA knockdown specifically in germ cells. (A)** Schematic illustration of reporter RNA knockdown by *Sycp1*-induced CRISPR-CasRx. Vector#3 carrying the *Sycp1* promoter encodes the Cre recombinase and non-targeting gRNAs (AAV9-*Sycp1*-Cre-NTG); Vector#4 carrying the *Sycp1* promoter encodes the Cre recombinase and tdTomato-targeting gRNAs (AAV9-*Sycp1*-Cre-tdTomato). The testes from a Cre-dependent CasRx knock-in mice (Rosa-CAG-LSL-CasRx-tdTomato-WPRE) were microinjected with AAV9-*Sycp1*-Cre-NTG or AAV9-*Sycp1*-Cre-tdTomato. **(B)** Representative stereomicroscopic fluorescent images of testes from CasRx-knock-in mice microinjected with AAV9-*Sycp1*-Cre-NTG or AAV9-*Sycp1*-Cre-tdTomato. Scale bar: 1 mm. **(C)** Mean fluorescence intensity (MFI) of tdTomato in testes of CasRx knock-in mice microinjected with AAV9-*Sycp1*-Cre-NTG or AAV9-*Sycp1*-Cre-tdTomato. Data were normalized to the tdTomato-non-targeting condition (*n* = 3). **(D and E)** Representative flow cytometry dot plots (D) and the corresponding quantification of the percentage of tdTomato^+^ cells (E) in the testes of CasRx knock-in mice microinjected with AAV9-*Sycp1*-Cre-NTG or AAV9-*Sycp1*-Cre-tdTomato. Data were normalized to the tdTomato-non-targeting condition (*n* = 3). All data are presented as the mean ± SEM. *P*-values were determined using the two-tailed Student's *t*-test; *P < 0.05; **P < 0.01.

We next aimed to test the ability of the *Sycp1* promoter to initiate the CRISPR-CasRx-mediated knockdown of RNA specifically in germ cells* in vivo*. We therefore constructed a tdTomato-targeting vector (AAV9-*Sycp1*-Cre-tdTomato) and the AAV9-*Sycp1*-Cre-NTG control, before microinjecting them into the testes of 3-week-old Cre-dependent CasRx knock-in mice (Rosa-CAG-LSL-CasRx-tdTomato-WPRE); in these assays, the CasRx was linked to tdTomato via a self-cleaving P2A peptide (Figure 6A). The knockdown efficiency of CasRx was determined by measuring germ-cell-specific tdTomato expression 3 weeks after microinjection. Fluorescence stereomicroscopy showed that the MFI of the testes treated with AAV9-*Sycp1*-Cre-tdTomato was visibly reduced (by ~60%) relative to that of the AAV9-*Sycp1*-Cre-NTG control (Figure 6B-C). Moreover, flow cytometry showed that the AAV9-*Sycp1*-Cre-tdTomato vector induced a ~5-fold decrease in the percentage of tdTomato^+^ germ cells relative to that in the control group (Figure 6D-E). Together, our results suggest that the *Sycp1*-promoter-initiating CRISPR-CasRx system effectively knocks down target genes in a germ-cell-type-specific manner.

## Discussion

In the present study, we optimized the *in vivo* AAV9-mediated delivery of CRISPR-CasRx into the testis and showed that the system induced effective exogenous and endogenous RNA depletion in this organ without adverse effects.

AAV has been identified as a versatile vehicle for transgene delivery because of its ability to transduce a variety of cell types, its lack of apparent pathogenicity, its ability to achieve long-term transgene expression without integrating into the host genome, and its overall safety 36. Previous reports have established that AAV9 preferentially infects Sertoli cells; however, its ability to transduce spermatogenic cell in the testis, particularly spermatids, remains limited 25. A possible explanation is that autophagy plays a protective role in spermatogenic cells. Autophagy is an intracellular catabolic process, which degrades and recycles proteins and organelles, thereby facilitating cellular homeostasis and survival 37. Autophagy also plays a significant role in host defense against microbial infection, including antiviral defense 38. As such, autophagy combats infections with RNA and DNA viruses by degrading viral components. Additionally, spermatogenesis itself is accompanied by increased levels of autophagy, which may potentially further reduce the efficiency of transducing spermatogenic cells with AAV9 39. Given these factors, we speculated that inhibiting autophagy during spermatogenesis may improve the AAV9 transduction efficiency of spermatogenic cells. To test this hypothesis, we used the common autophagy inhibitor 3-MA to show that inhibiting autophagy indeed improved the *in vivo* AAV9 transduction efficiency of spermatogenic cells. Additional experiments will be required to understand the mechanisms underlying this 3-MA-mediated improvement in AAV9 transduction efficiency, which could also be applicable to organs other than the testis.

Long-term gene modulation and safety are important considerations for future clinical investigation of AAV-mediated transgene delivery. Hence, it was essential to study the transduction kinetics, organ distribution, and safety of AAV9 and 3-MA co-administration *in vivo*. Our findings demonstrated that AAV9-mediated transgene expression could be detected at 1 week and peaked at 3-4 weeks after microinjection. Additionally, AAV9 and 3-MA co-injection induced efficient and long-term gene expression (> 15 weeks) in the testis, which followed a rhythmic expression pattern. We surmised that this 5-weekly cycling of gene expression synchronized with the spermatogenic wave of mice. Importantly, the co-injection of AAV9 and 3-MA did not substantially influence the integrity of the BTB or the testicular microenvironment. These results suggested that AAV9 and 3-MA could be used efficiently and safely to deliver transgenes into the testis *in vivo*.

Spermatogenesis is a tightly controlled process, which relies on the precise regulation of specific transcriptional programs at each stage of development 40. Previous studies have proposed the role of lncRNAs in spermatogenesis and emphasized the significance of experimentally disrupting RNA activity *in vivo* as a means of understanding their biological functions in this process 41, 42. Despite these promising findings, the functional roles of many lncRNAs in spermatogenesis remain unclear, partly due to the lack of effective tools to perturb lncRNA activity (and particularly that of nuclear lncRNAs) in the testis *in vivo*. The CRISPR-CasRx system provides a highly specific and efficient method to deplete specific RNA transcripts in plants, *Drosophila*, mammalian cell lines, as well as in murine neurons, liver, and embryos 8, 16, 24, 43, 44. Despite the broad applicability of the CRISPR-CasRx system, the ability to perturb RNA function in the testis *in vivo* requires further evaluation. In this study, we generated an all-in-one AAV delivery vehicle comprising a gRNAs array and an mRNA encoding the CasRx protein, and demonstrated that AAV9-mediated CRISPR-CasRx induced the degradation of specific RNA transcripts, which exhibits superior RNA knockdown efficiency and specificity to those of RNAi in the testis *in vivo*. To rule out the possibility that RNA transcript depletion arose as a result of decreased cell numbers, caused by the disruption of functional gene expression, we tested the knockdown efficiency and specificity of CasRx for a tdTomato fluorescence reporter transiently overexpressed in testis *in vivo*. The rationale for selecting tdTomato was that, theoretically, its expression would not have a significant effect on cell maintenance. We showed that the AAV9-mediated CRISPR-CasRx system successfully knocked down tdTomato reporter mRNA expression without significantly altering the expression levels of typical functional genes in the testis. Furthermore, by targeting the nuclear lncRNA-*Eif2c5*, we established that the CRISPR-CasRx system could effectively knock down both cytoplasmic and nuclear RNAs and was therefore a useful tool for studying gene function *in vivo* during germline development. In this regard, the AAV9-mediated CRISPR-CasRx system is a promising molecular tool for transcriptome engineering and offers several benefits when used in the testis *in vivo*. For example, the versatility and simplicity of this tool enable the large-scale screening of functional RNAs involved in spermatogenesis before investing considerable effort in investigating the molecular functions of a select few candidates in a knockout animal model. Moreover, local RNA knockdown but not permanent DNA knockout is ideal for studying target genes, the deletion of which may lead to early embryonic lethality, as well as minimizing multiple off-target effects which could result from the deletion of an entire gene locus (e.g., in the case of lncRNAs). Moreover, the robust RNA cleavage property of CRISPR-CasRx makes it a highly promising method for the treatment of testicular infections caused by single-stranded RNA viruses, such as severe acute respiratory syndrome coronavirus 2 (SARS-CoV-2) or Zika virus. Infections with these RNA viruses may induce inflammation, damage testicular tissue, and perturb spermatogenesis 45-47. Recently, CRISPR-Cas13 has been shown to effectively interfere with multiple SARS-CoV-2 variants *in vitro* 48. Our study validated the fact that AAV9-mediated CRISPR-CasRx effectively targeted RNA transcripts in the testis *in vivo*, further showing the potential of CRISPR-CasRx in RNA-targeting therapies.

The cell-type-specific activation of the CRISPR system has been an ongoing challenge, due to the need to ensure both the safety and efficacy of its *in vivo* application 49. One strategy for achieving germ-cell-type-specific, CRISPR-mediated gene editing involves employing a germ-cell-specific gene promoter. We selected four genes, *Stra8*,* Hspa2*, *Sycp1* and *Pgk2*, for the screening of germ-cell-specific promoters. Among them, the promoter of *Stra8*, *Hspa2* and *Pgk2* genes induced RFP expression in both germ and Sertoli cells. We tend to believe that such result was due to a partial promoter fragment instead of the complete promoter sequence as well as absence of the negative *cis* element, which led to non-specific expression of initiating RFP gene. Our work revealed that the promoter of a germ-cell-specific gene, *Sycp1,* successfully drove transgene expression in meiotic germ cells *in vivo*. Moreover, we showed that an AAV9 vector carrying the *Sycp1* promoter was able to maintain long-term transgene expression (up to 3 weeks) in the testis *in vivo*. The ability to efficiently induce target gene knockdown in a germ-cell-type-specific manner underscores the potential of this approach as a therapeutic application *in vivo*.

In summary, we have optimized the AAV9-mediated delivery of the CRISPR-CasRx system to the testis and demonstrated the feasibility of this approach in efficiently and safely knocking down endogenous and exogenous RNA expression in the testis *in vivo*. Moreover, we achieved germ-cell-specific, CRISPR-CasRx-mediated RNA knockdown by including the *Sycp1* promoter in our vector construct. We envisage that these developments will facilitate transcriptome-related investigations and the development of RNA-targeting therapies for reproduction health conditions.

## Methods

### Plasmid construction

All AAV expression vectors used for packaging in AAV9 were constructed using an AAV-based cloning vector backbone (pAAV.H1.CMV.RFP). To construct the all-in-one AAV expression vector for the pre-gRNAs array and the mRNA-encoding CasRx protein (AAV9-EFS-CasRx-U6-gRNA), the NLS-CasRx-NLS and DNA sequences were PCR-amplified from the plasmids expressing CasRx (Addgene, #109049) or pre-gRNA scaffold (Addgene, #109054), respectively. These cassettes were then cloned into an AAV-based cloning vector using single-step Gibson assembly with the NEBuilder^®^ HiFi DNA Assembly Master Mix (NEB, Cat# E2621L), according to the manufacturer's instructions. The sequences of oligonucleotides used to construct the gRNAs array were synthesized by Beijing Tianyi Huiyuan Biotechnology and then cloned into the linearized AAV9-EFS-CasRx-U6-gRNA plasmid using the BplI restriction site. The gRNAs were designed using the cas13design tool 18. The sequences of gRNAs used in this study are provided in Table S1.

To construct the AAV reporter plasmids carrying the germ-cell-specific gene promoter fused to an RFP reporter (AAV9-*Stra8*-RFP, AAV9-*Hspa2*-RFP, AAV9-*Sycp1*-RFP, and AAV9-*Pgk2*-RFP), the following promoter fragments were PCR-amplified from mouse genomic DNA: -772/+102 of *Sycp1*, -371/+29 of *Stra8*, -932/-225 of *Hspa2*, and -515/0 of *Pgk2*. Next, these fragments were individually cloned into the AAV-based cloning vector using single-step Gibson assembly. The primer sequences for the germ-cell-specific gene promoters are listed in Table S2.

To construct the AAV expression vector AAV9-*Sycp1*-Cre-U6-gRNAs, the U6-DRs-gRNAs DNA sequence was PCR-amplified from the plasmid expressing the pre-gRNA scaffold (Addgene, #109054); meanwhile, the cyclization recombination enzyme (Cre) DNA sequence was PCR-amplified from the genomic DNA of the *Cre*-transgenic mouse. These cassettes were then cloned into the linearized AAV9-*Sycp1*-RFP plasmid via BamHI and XhoI using single-step Gibson assembly. The primer sequences used for *Cre* amplification are listed in Table S2.

### Cell culture and transfections

HEK293T cells and GC1-spg cells were obtained from the Cell Resource Center, Peking Union Medical College. HEK293T cells and GC1-spg cells were cultured in Dulbecco's modified Eagle's medium (DMEM) (Corning) containing 10% fetal bovine serum (FBS), supplemented with 1% penicillin-streptomycin, in a humidified 5% CO_2_ incubator at 37 °C.

To determine the knockdown efficiency of the tdTomato reporter, *Sycp3*, and lncRNA-*Eif2C5*, mediated by CRISPR-CasRx, an average of 5 × 10^5^ HEK293T cells were seeded into 6-well plates and transfected with 1 μg of AAV expression vector expressing the target gene and 1.5 μg of the all-in-one AAV CRISPR-CasRx expression vector (AAV9-EFS-CasRx-U6-gRNA). Transfection was achieved using Lipofectamine 3000 (Thermo Fisher Scientific) according to the manufacturer's protocol. The transfected cells were harvested 48 h post-transfection and subjected to qRT-PCR, western blotting, and fluorescence imaging.

To determine the knockdown efficiency of lncRNA-*Malat1* by CRISPR-CasRx, an average of 5 × 10^5^ GC1-spg cells were seeded into 6-well plates and infected with a lentivirus expressing CasRx and gRNAs targeting lncRNA-*Malat1*. GC1-spg cells were harvested 48 h post-infection and sorted by flow cytometry for qRT-PCR analysis.

### AAV9 packaging

AAV-HEK293 cells (HEK293 cells stably expressing the E1 gene region) were obtained from the Cell Resource Center. AAV-HEK293 cells were cultured in DMEM containing 10% FBS supplemented with 1% penicillin-streptomycin in a humidified 5% CO_2_ incubator at 37 °C. AAV-HEK293 cells were plated in 150-mm culture dishes and grown to reach 70% confluency. The AAV vector plasmids, pAAV2/9, and the adenoviral helper plasmids, were transfected at a ratio of 1:1:1 in serum-free Opti-MEM (Gibco, Cat# 31985070) using PEI Max at a DNA:PEI ratio of 1:2. The culture cells and supernatant were collected 120 h after transfection. Subsequently, the crude AAV9 fraction was treated with 50 units/mL UltraNuclease (YEASEN, Cat# 20156) for 30 min at 37 °C, and then with 5 M NaCl at 50 °C for 30 min. The pellet debris was then removed by centrifugation at 12,000 ×g for 30 min. Next, AAVs were purified by gradient centrifugation using 9, 6, 5, and 5 mL of 15%, 25%, 40%, and 54% iodixanol, respectively. After ultracentrifugation at 350,000 ×g for 90 min, 4 mL of the transparent layer were extracted into PBS and the viral particles were obtained. AAV titers (GC/mL) were determined by qRT-PCR. After viral titration (>10^13^ GC/mL), the AAV was aliquoted and stored at -80 °C until used.

### Animals and microinjection

For the *in vivo* experiments, male CD-1/ICR mice were purchased from Beijing Sibeifu Biotechnology Co. Ltd. Ai9 mice (Rosa26-CAG-LSL-tdTomato) were obtained from the Shanghai Model Organisms Center. CasRx knock-in mice (Rosa-CAG-LSL-CasRx-tdTomato-WPRE) were a gift from Professor Yu Nie, Peking Union Medical College 50. Male mice (3-week-old) were used in the AAV9 or siRNA microinjection experiments performed in this study, unless otherwise specified. Microinjection was conducted as described in our previous study 14. Briefly, ~10 µL of viral particles containing 0.04% Trypan blue were injected via the efferent duct into the seminiferous tubules. To improve transduction efficiency of AAV9, 3-MA (at a final concentration of 10 mM) was co-injected with the virus. A dual-virus model was employed to confirm the knockdown efficiency and specificity of the CRISPR-CasRx system for the exogenous reporter tdTomato. The dual-viral particle ratio of AAV9-EFS-tdTomato to AAV9-EFS-CasRx-NTG or AAV9-EFS-CasRx-tdTomato was 1:2. For siRNA microinjection, siRNA pool (Ribobio, Guangzhou, China) chemically modifed with 2'-OM (2-methoxyethyl) and cholesterol was used for the exogenous reporter tdTomato knockdown:

tdTomato siRNAs (target sequences): 5'-AGCGCGTGATGAACTTCGA-3', 5'-AAGAGTTCATGCGCTTCAA-3', 5'-CACGAGTTCGAGATCGAGG-3'.

Microinjection of siRNA (100 µM) was operated as the CRISPR-CasRx condition. The ratio between AAV9-EFS-tdTomato and siRNA pool was 1:2. All experimental procedures were approved by the Animal Experimental Ethical Inspection of Institute of Basic Medical Sciences, Chinese Academy of Medical Sciences and Peking Union Medical College.

### Tracer experiment

The tracer experiment was performed as previously described 25. Approximately 15 μL Sulfo-NHS-LC-biotin (7.5 mg/mL; Thermo Fisher Scientific, Cat# A39257) was interstitially injected into the testis 3 weeks after the AAV injection. The mice were euthanized 30 min post-injection and their testes were immediately collected, and fixed in 4% paraformaldehyde (PFA) overnight at 4 °C. The testis samples were then embedded in the Tissue-Tek OCT compound (Sakura Finetek, Cat# 4583) for 7-μm-thick cryosections. The sections were incubated with Alexa Fluor 488-conjugated streptavidin (Thermo Fisher Scientific, Cat# S11223) and counterstained using Fluoroshield mounting medium (Sigma-Aldrich, Cat# F6057). Fluorescent images were captured using a Zeiss LSM780 confocal microscope.

### *In vivo* animal imaging

*In vivo* image acquisition and analysis were performed weekly after the AAV injections using the Xenogen IVIS spectrum imaging system. Each mouse was anesthetized with 2% isoflurane and placed in the prone position in the bioluminescent imager. The fluorescent intensity was measured and quantified at different time-points. Spectral unmixing was used for image reconstruction. The region of interest (ROI), encompassing the whole testis, was manually outlined on the fused images for use in further analyses of radiant efficiency and to compare different time-points. Each mouse was imaged on a weekly basis for up to 15 weeks after AAV microinjection.

### RNA extraction and quantitative real-time PCR (qRT-PCR)

Total RNA was extracted from the testes 3-4 weeks after AAV microinjection using the TRIzol reagent (Thermo Fisher Scientific, Cat# 15596018) according to the manufacturer's instructions. cDNA was synthesized using the RevertAid First Strand cDNA Synthesis Kit (Thermo Fisher Scientific, Cat# K1622) and processed for qRT-PCR using the PerfectStart® Green qPCR SuperMix (TransGen, Cat# AQ601) on the CFX Connect System. Relative expression levels of target genes were quantified using the delta-delta *Ct* (*ΔΔCt*) method. The data were normalized to *Gapdh* or *Actb* expression. Primer sequences used in this study are listed in Table S3.

### Western blotting

The samples lysates were prepared in 1 × sodium dodecyl sulfate buffer supplemented with protease inhibitor cocktail. Protein concentration was measured using the Pierce BCA Protein Assay Kit (Thermo Fisher Scientific, Cat# 23227). Protein samples were separated by SDS-PAGE electrophoresis using 10% polyacrylamide gels (EpiZyme Biotechnology, Cat# PG112) and then transblotted onto polyvinylidene difluoride (PVDF) membranes. After blocking with 5% skimmed milk for 1 h at room temperature (RT), the membranes were incubated with the primary antibodies overnight at 4 °C. After a washing step with 1× TBST, the membranes were incubated with horseradish peroxidase (HRP)-conjugated secondary antibody for 1 h at RT. Protein blots were visualized using the Fusion FX EDGE chemiluminescence imaging system.

### RNA sequencing

Sequencing libraries were generated using the VAHTS mRNA-seq v2 Library Prep Kit for Illumina by following manufacturer's recommendations. Next, library quantification was performed using the Qubit high sensitivity assay, while quality control was conducted using the Agilent 2100 bioanalyzer or Fragment Analyzer 5300; the final library has an average size of ~350 bp. The libraries were sequenced on an Illumina NovaSeq platform to generate 150-bp paired-end reads. Sequence reads were mapped to the mouse genome (version mm10) using the HISAT2 (v2.0.0). Differentially expressed genes were defined as those with an adjusted *P*-value (*P*_adj_) < 0.05 and a log_2_ fold change (log_2_FC) > 2 using the DEGSeq R package (v1.20.0).

### Flow cytometry

Testicular cells were isolated from the testes received microinjection of AAV9 as previously described 14, 51. In brief, the testes were decapsulated and incubated with collagenase type IA (0.5 mg/mL) at 37 °C for 15 min to separate the interstitial cells and seminiferous tubules. After a mincing step, the seminiferous tubules were digested with collagenase type IV (1 mg/mL) and DNase I (500 μg/mL) at 37 °C for 15 min with gentle oscillation. The collagenase digestion process was stopped using DMEM containing 10% FBS. For germ cell isolation, the testicular cells were cultured in F12/DMEM containing 10% FBS for 6 h at 37 °C and then recovered by collecting nonadherent cells. The cell suspensions were then filtered through a 40-mm nylon mesh and re-suspended in PBS containing 2% FBS for flow cytometry analysis and RFP or tdTomato-positive cell sorting on a MA900 Multi-Application Cell Sorter (SONY). A total of 10^5^ events were recorded per sample and the data were analyzed using Kaluza Analysis Software (BECKMAN).

### Immunofluorescence staining and the RNAscope in situ hybridization assay

Testes from AAV9-microinjected mice were dissected, and fixed in 4% PFA overnight at 4 °C. After cryoprotection in sucrose-PBS solution (10% sucrose for 1 h, 20% sucrose for 1 h, and 30% sucrose overnight) at 4 °C the testes were embedded in the Tissue-Tek OCT compound and sectioned into 7-μm-thick cryosections for subsequent staining. After a blocking step in 5% bovine serum albumin with 0.1% Triton X-100 for 1 h at RT, the cryosections were incubated with primary antibodies overnight at 4 °C. After washing with PBS, the sections were incubated with an AlexaFluor™-conjugated secondary antibody for 1 h at RT. Subsequently, the sections were mounted using Fluoroshield mounting medium (Sigma-Aldrich, Cat# F6057) and the fluorescent images were visualized using a confocal microscope (LSM780). Number of cells with staining were calculated using image analysis software Image J. The primary antibodies and secondary antibodies used in this study are listed in Table S4.

For RNAscope *in situ* hybridization, the Multiplex Fluorescent Manual Assay Kit and RNAscope^®^ Probe-Ms-*Eif2c5* were purchased from Advanced Cell Diagnostics (ACD; Hayward, CA, USA). The pretreatment of testicular sections, target retrieval, protease plus treatment, target probe hybridization, signal amplification, counterstaining, and mounting were performed according to the manufacturer's instructions. Fluorescent signals were detected on a Zeiss LSM780 confocal microscope. Quantification of *Eif2c5* punctate signal per tubule were calculated using image analysis software Image J.

### Statistical analysis

Statistical analysis was performed using GraphPad Prism 7. All experiments were independently repeated at least three times, and experimental data were represented as mean ± SEM. Statistically significant differences (*P* < 0.05) were assessed using two-tailed Student's *t*-tests as indicated in figures and figure legends.

## Supplementary Material

Supplementary figures and tables.
