# Supplementary material for: Adeno-associated-virus-mediated delivery of CRISPR-CasRx induces efficient RNA knockdown in the mouse testis
Source: Theranostics. 2024 Jun 17;14(10):3827–42. doi: 10.7150/thno.95633 (PMC11234267; doi:10.7150/thno.95633)
Supplement: Supplementary file 1 — Supplementary figures and tables. [file thnov14p3827s1.pdf]

Supporting Information

Adeno-associated-virus-mediated delivery of CRISPR-CasRx induces efficient RNA knockdown in the mouse testis

Kai Li†, Mengzhen Li†, Yanyun Luo†, Dingfeng Zou, Yahui Li, Xinyu Mang, Zexuan Zhang, Pengyu Li, Yan Lu, Shiying Miao & Wei Song*

Department of Biochemistry and Molecular Biology, State Key Laboratory of Common Mechanism Research for Major Diseases, Institute of Basic Medical Sciences, Chinese Academy of Medical Sciences and Peking Union Medical College, Beijing 100005, China.

†These authors contributed equally to this work
* Corresponding author (Wei Song, email: songwei@ibms.pumc.edu.cn)

Contents:

Supplemental Figures S1-S4

Supplemental Tables S1-S4

SF1

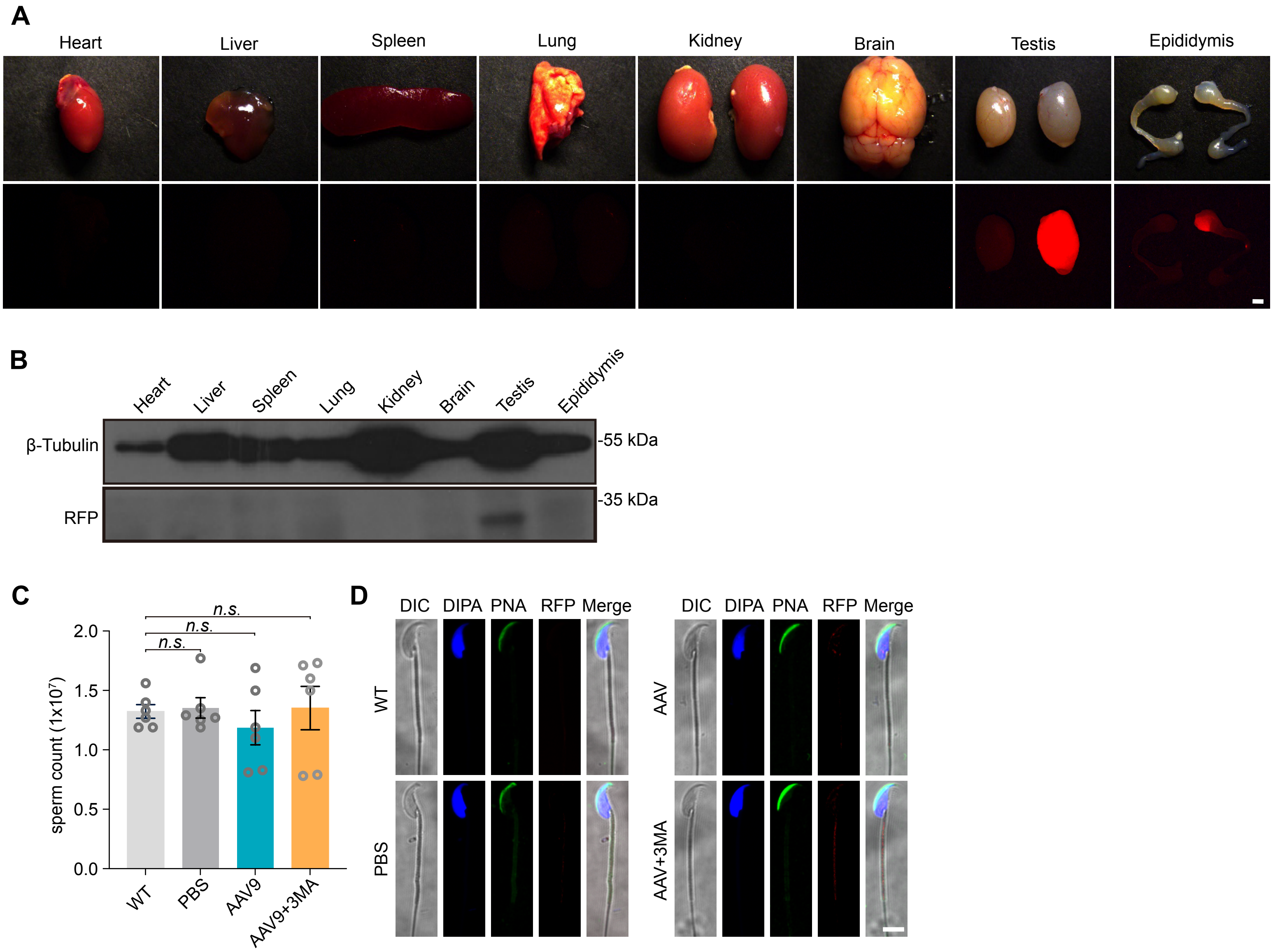

A
Heart
Liver
Spleen
Lung
Kidney
Brain
Testis
Epididymis
B
Heart
Liver
Spleen
Lung
Kidney
Brain
Testis
Epididymis
β-Tubulin
RFP
-55 kDa
-35 kDa
C
n.s.
n.s.
n.s.
sperm count (1x10⁷)
2.0
1.5
1.0
0.5
0.0
WT
PBS
AAV9
AAV9+3MA
D
DIC
DIPA
PNA
RFP
Merge
WT
PBS
AAV
AAV+3MA

Figure S1. Expression profiling of transgenic tissues and developmental assessment of spermatozoa after AAV microinjection into testicular seminiferous tubules.
(A) Stereomicroscopic fluorescence imaging of various tissues from mice co-injected with AAV9-CMV-RFP and 3-MA. Scale bar: 1 mm.
(B) Western blotting analysis of RFP in various tissues from mice co-injected with AAV9-CMV-RFP and 3-MA. β-Tubulin was used as a loading control.
(C) Number of mature sperm from representative caudal epididymis 5 weeks after microinjection with PBS, AAV9-CMV-RFP or AAV9-CMV-RFP co-injection with 3-MA ($n = 5$).
(D) Immunostaining of spermatozoa for PNA(green) ,RFP(red) from representative caudal epididymis 5 weeks after microinjection with PBS, AAV9-CMV-RFP or AAV9-CMV-RFP co-injection with 3-MA. Nuclei were stained with DAPI (blue). Scale bar: 5 μm.
All data are presented as the mean ± SEM. *P*-values were determined using the two-tailed Student's t-test; $^{n.s.}$ $P > 0.05$.
.

Figure S2

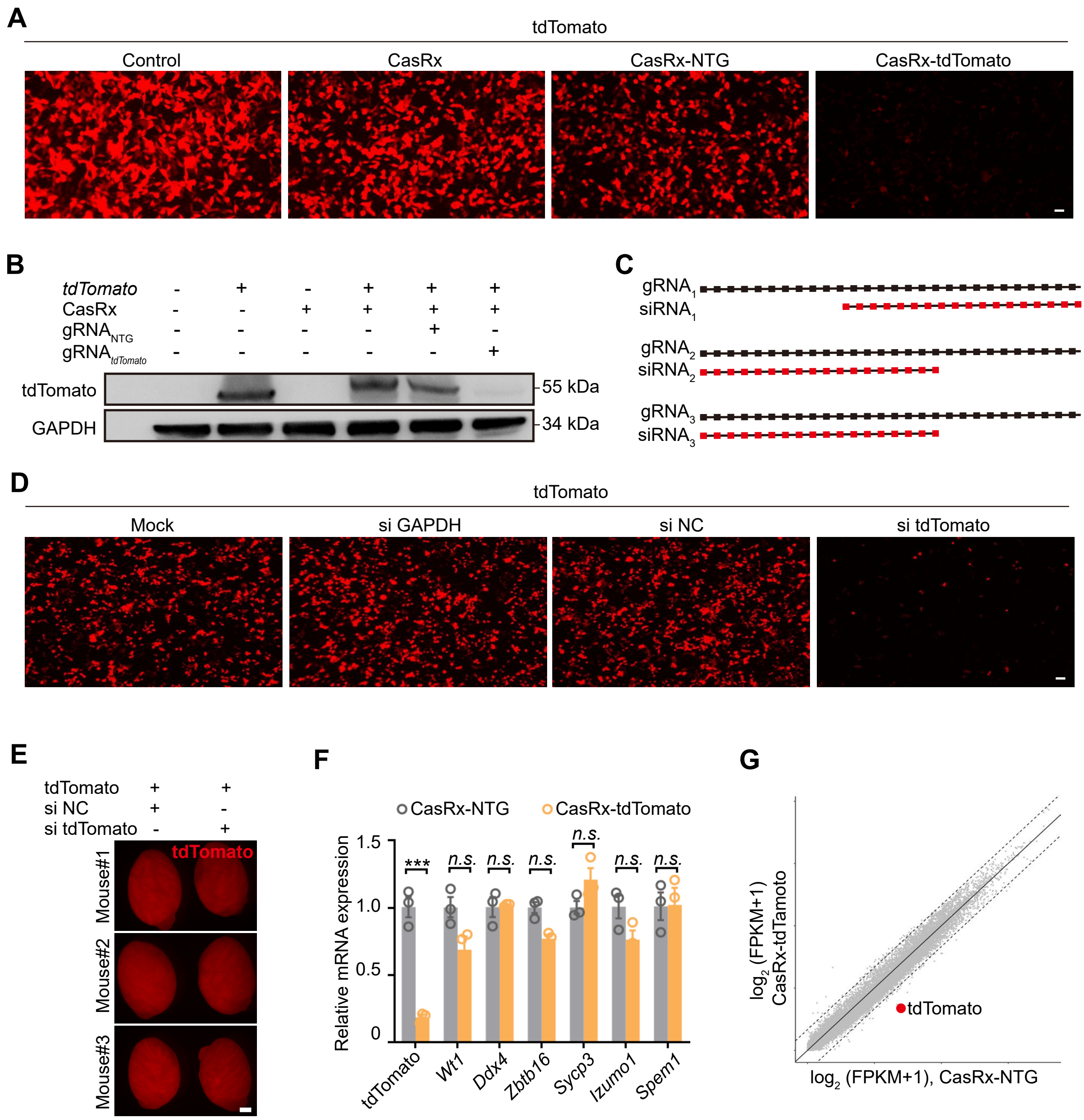
A
tdTomato
Control
CasRx
CasRx-NTG
CasRx-tdTomato
B
tdTomato
CasRx
gRNA NTG
gRNA tdTomato
tdTomato
GAPDH
55 kDa
34 kDa
C
gRNA1
siRNA1
gRNA2
siRNA2
gRNA3
siRNA3
D
tdTomato
Mock
si GAPDH
si NC
si tdTomato
E
tdTomato
si NC
si tdTomato
tdTomato
Mouse#1
Mouse#2
Mouse#3
F
CasRx-NTG
CasRx-tdTomato
Relative mRNA expression
1.5
1.0
0.5
0
***
n.s.
tdTomato
Wt1
Ddx4
Zbtb16
Sycp3
Izumo1
Spem1
G
log2 (FPKM+1) CasRx-tdTamoto
log2 (FPKM+1), CasRx-NTG
tdTomato

Figure S2. CRISPR-CasRx enables efficient reporter mRNA knockdown in HEK293T cells.

(A) Fluorescence imaging of tdTomato in HEK293T cells transfected with an expression vector encoding the tdTomato reporter as well as an all-in-one vector encoding CasRx and gRNAs. Scale bar: 40 μm.

(B) Western blotting analysis of tdTomato expression in HEK293T cells after CRISPR-CasRx gene editing. GAPDH was used as a loading control.

(C) Schematic drawing of tdtomato-targeting guide RNAsequences and spacers position matched siRNA.

(D) Fluorescence imaging of tdTomato in HEK293T cells transfected with expression vector encoding tdTomato reporter as well as siRNAs. Scale bar: 40 μm.

(E) Stereomicroscopic fluorescence imaging of representative testes received either microinjection with siRNA non-targeting or siRNA tdtomato-targeting together with AAV9-EFS-tdTomato. Scale bar: 1 mm.

(F) qRT-PCR analysis of representative functional genes expression in somatic and spermatogenic cells. Data were normalized to *Actb* expression (n = 3 biologically independent samples).

(G) Scatterplot showing mRNAlevels (determined by RNA-seq) in testes microinjected with AAV9-EFS-CasRx-tdTomato (y-axis) relative to those in the control testes treated with AAV9-EFS-CasRx-NTG (x-axis). tdTomato mRNAis highlighted in red. Dashed lines indicate a 2-fold difference in RNA levels (n = 3 biologically independent samples).

Figure S3

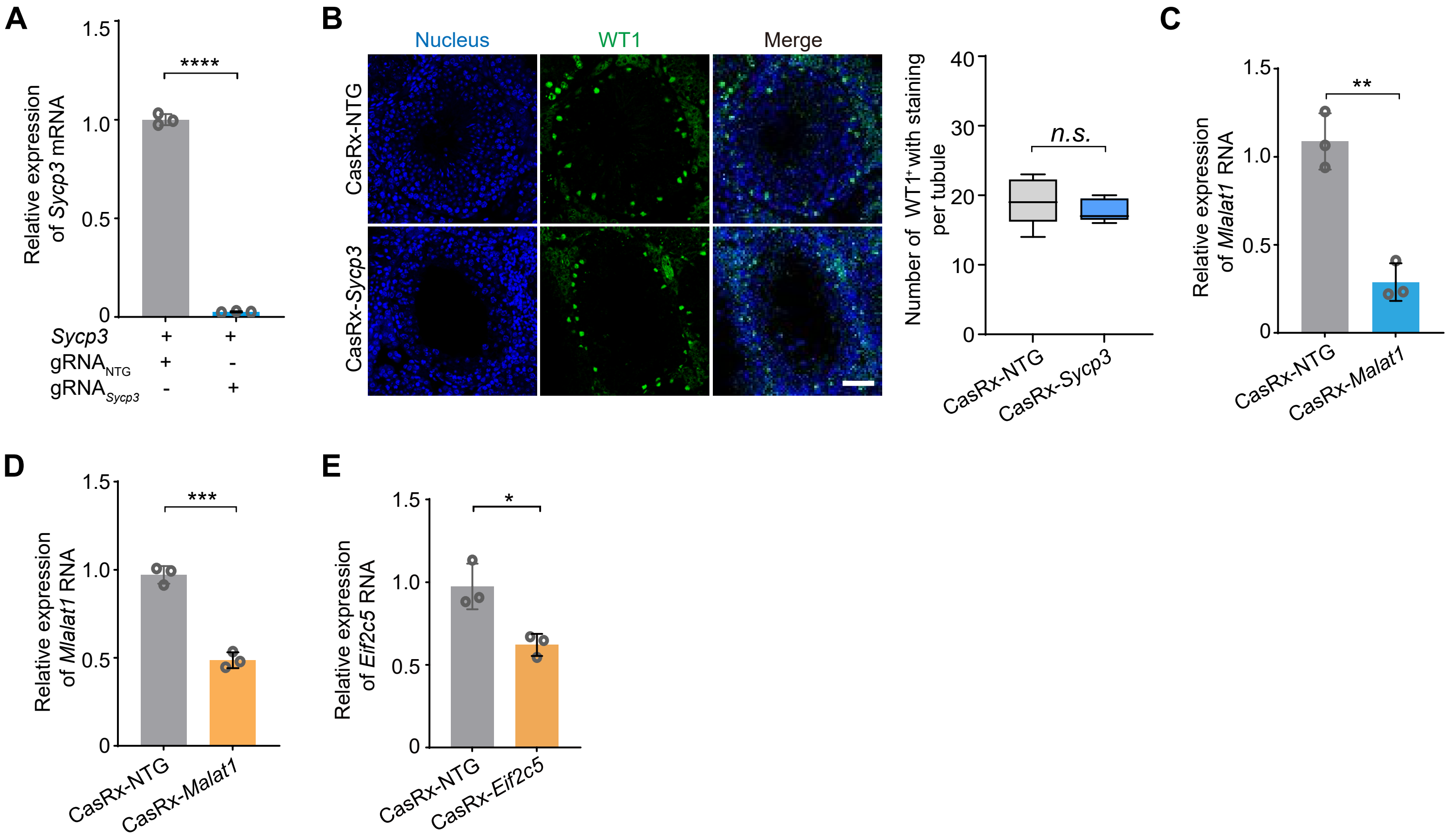
A
Relative expression of Sycp3 mRNA
1.5
1.0
0.5
0
****
Sycp3 + +
gRNANTG + -
gRNASycp3 - +
B
Nucleus
WT1
Merge
CasRx-NTG
CasRx-Sycp3
Number of WT1+ with staining per tubule
40
30
20
10
0
n.s.
CasRx-NTG
CasRx-Sycp3
C
Relative expression of Mlalat1 RNA
1.5
1.0
0.5
0
**
CasRx-NTG
CasRx-Malat1
D
Relative expression of Mlalat1 RNA
1.5
1.0
0.5
0
***
CasRx-NTG
CasRx-Malat1
E
Relative expression of Eif2c5 RNA
1.5
1.0
0.5
0
*
CasRx-NTG
CasRx-Eif2c5

Figure S3. Transcriptome editing of *Sycp3* and lncRNA-*Eif2c5* by AAV9-mediated CRISPR-CasRx in the testis *in vivo*.

(A) qRT-PCR analysis of *Sycp3* mRNA knockdown efficiency in HEK293T cells transfected with the expression vector encoding SYCP3 as well as the all-in-one vector encoding CasRx and gRNAs. Data were normalized to *Actb* expression (n = 3 biologically independent samples).

(B) Immunostaining of testicular sections from the control (CasRx-NTG) and SYCP3-depleted (CasRx-*Sycp3*) mice for WT1 (green). Nuclei were stained with DAPI (blue). Scale bar: 50 μm. *Left*: representative images; *Right*: number of stained cells per tubule (n = 10).

(C) qRT-PCR analysis of lncRNA-*Malat1* knockdown efficiency in GC1 cells. Data were normalized to *Actb* expression (n = 3 biologically independent samples).

(D) qRT-PCR analysis of lncRNA-*Malat1* knockdown efficiency targeted by AAV9-mediated CRISPR-CasRx *in vivo*. Data were normalized to *Actb* expression (n = 3 biologically independent samples).

(E) qRT-PCR analysis of lncRNA-*Eif2c5* knockdown efficiency *in vivo*. Data were normalized to *Actb* expression (n = 3 biologically independent samples).

All data are presented as the mean ± SEM. P-values were determined using the two-tailed Student's t-test; $^{n.s.}P > 0.05$; $^{*}P < 0.05$; $^{**}P < 0.01$; $^{***}P < 0.001$; $^{****}P < 0.0001$.

Figure S4

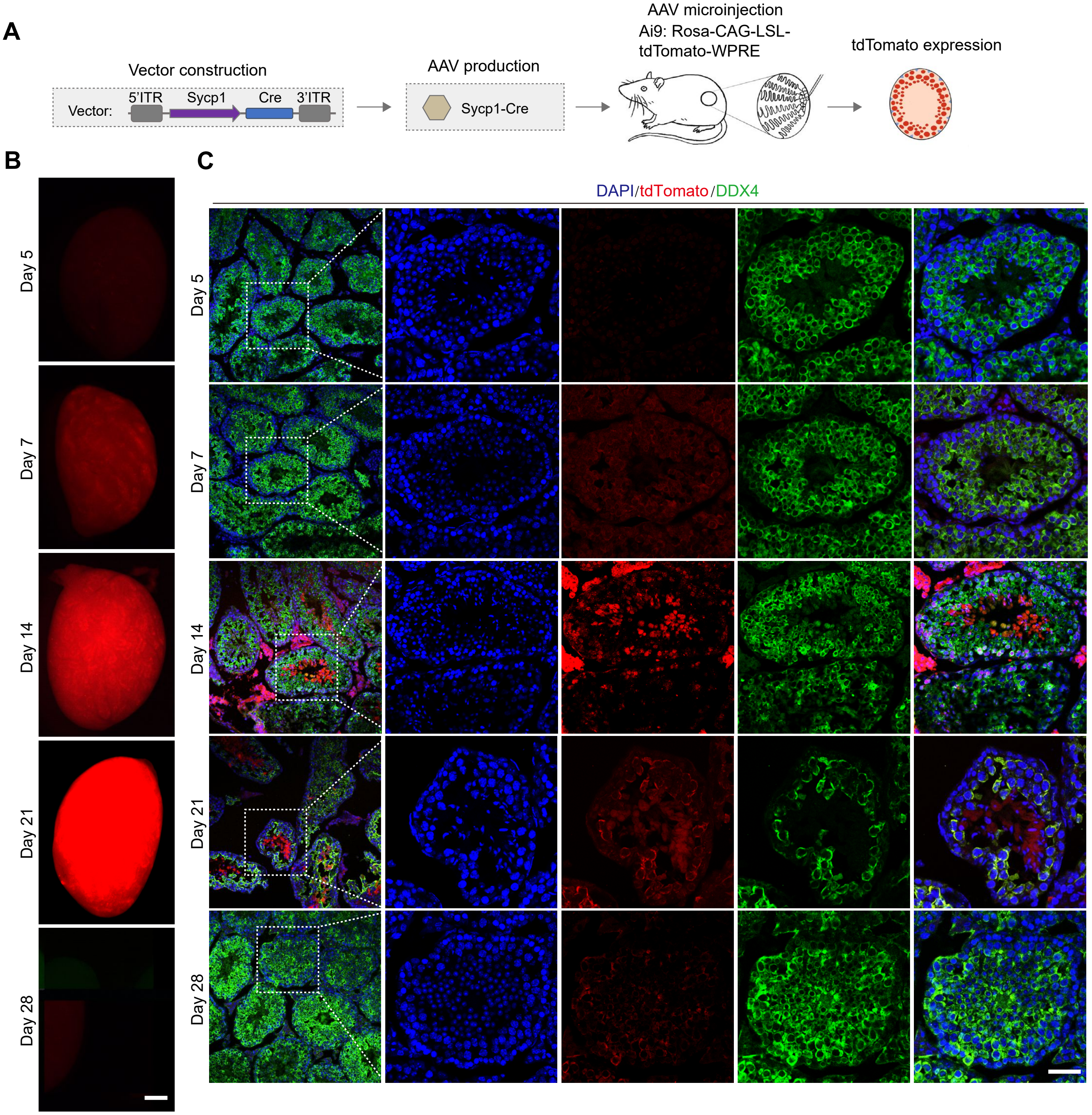
A
Vector construction
Vector:
5'ITR
Sycp1
Cre
3'ITR
AAV production
Sycp1-Cre
AAV microinjection
Ai9: Rosa-CAG-LSL-tdTomato-WPRE
tdTomato expression
B
Day 5
Day 7
Day 14
Day 21
Day 28
C
DAPI/tdTomato/DDX4
Day 5
Day 7
Day 14
Day 21
Day 28

Figure S4. AAV9 directs the Sycp1 promoter to drive transgene expression in germ cells *in vivo*.
(A) Schematic illustration of how AAV9-mediated germ-cell-specific transgene expression is driven by the *Sycp1* promoter. A vector carrying the *Sycp1* promoter and encoding the Cre recombinase is packaged into AAV9 (AAV9-*Sycp1*-Cre) and microinjected into the testes of an Ai9 mouse (Rosa26-CAG-LSL-tdTomato).
(B) Representative stereomicroscopic fluorescent images of testes from Ai9 mice on days 5, 7, 14, 21, and 28 after microinjection of AAV9-*Sycp1*-Cre. Scale bar: 1 mm.
(C) Immunostaining of testicular sections from Ai9 mice on days 5, 7, 14, 21, and 28 after microinjection of AAV9-*Sycp1*-Cre for DDX4 (green) and tdTomato (red). Nuclei were stained with DAPI (blue). Scale bar: 50 μm.

Supplemental Tables

Table S1. gRNA sequences used for knockdown of mRNA and lncRNA
Table S2. Primer sequences of PCR
Table S3. Primer sequences of qRT-PCR
Table S4. Antibodies used for immunofluorescence

Table S1. gRNA sequences used for knockdown of mRNA and lncRNA

	gRNA sequences 1# (5' to 3')	gRNA sequences 2# (5' to 3')	gRNA sequences 3# (5' to 3')
tdTomato	CATGCGCACCTTGAAGCGC ATGAACTCTTT	ATGGCCGTCATCAAAGAGT TCATGCGCTTC	TACGTGGACACCAAGCTGG ACATCACCTCC
Sycp3	AACATCTTCAATTATCCCA GCAGATCTTTT	CCCAGAATGCTTTCTTCCAC CAGGCACCAT	AATTTCTGTATATCCAGTTC CCACTGCTGC
Malat1	CCAGATGTTAAAACAAGCC CAGGGCCTCTC	AACATTTACCTAAGGCAGC ACAGCAAAGGG	TCGTTTTAATCTACAAGGC CGACCTTCAAA
Eif2c5	TAATACACTCGAAGTACCA GCAGGTCCCAC	ATACCTTTTAAAGTACGGC ACATACAGTTG	CAGATGATAGATAATGGAA CCAGGCTTTTG
NTG	TCACCAGAAGCGTACCATA CTCACGAACAG	CTACCTGGTAGCCCTTGTAT TTGATCAGGC	TGCCACTACTGTTCATGATC AGGGCGATGG

Table S2. Primer sequences of PCR

	Forward primer (5' to 3')	Reverse primer (5' to 3')
NLS-Cas13d - NLS	TTTGCCGCCAGAACACAGGACCGGTGCC ACCATGAGCCCCAAGAAGAAG	TTATCATGTCTGCTCGAAGCGGCCTTAA GCGTAATCTGGAACATCGTATGGGTAAG CG
U6-DRs-gRNAs (Vector2#)	CAAATGTGGTAAAATCGAGAGCATGGC TACGAGGGCCTATTTCCCATGATTCCTT CAT	TGATTAACCCGCCATGCTACTTATCTAC ATGCGTAAGGAGAAAATACCGCATCAG A
Stra8 promoter	GATCAATTCAATTCACGCGTATCCCCTA TTCCCCTCTC	CATGGTGGCACTAGGCTAGCTCTAGAGG GATCCCCGTCGCAGA
Hspa2 promoter	GATCAATTCAATTCACGCGTCGGTCGTT CTACATTACAGGTC	GGTGGCACTAGGCTAGCGGGATCCCCTG CCTGCTG
Sycp1 promoter	GATCAATTCAATTCACGCGTATCTTGTC CTAGGTATTAAAACAGG	CATGGTGGCACTAGGCTAGCGGGATCC GGGAGGCT
Pgk2 promoter	GATCAATTCAATTCACGCGTTCACAAAG TCTAATAGCAGATCA	GCACTAGGCTAGCGGGATCCTTGGCCCC GCCTTTCTTTGTGAG
Cre	TCCCGCTAGCCTAGTGCCACCATGCCCA AGAAGAAGAGGAAGGTGTCCAATT	AGCGTAATCTGGAACATCGTATGGGTAA GCGGCCGCATCGCCATCTTCCAGCAGGC GCACCATTG
U6-DRs-gRNAs (AAV-*Sycp1*-Cre-U6-gRNAs)	CAAATGTGGTAAAATCGAGAGCATGGC TACGAGGGCCTATTTCCCATGATTCCTT CAT	TGATTAACCCGCCATGCTACTTATCTAC ATGCGTAAGGAGAAAATACCGCATCAG A

Table S3. Primer sequences of qRT-PCR

	Forward primer (5' to 3')	Reverse primer (5' to 3')
Actb	GGCTGTATTCCCCTCCATCG	CCAGTTGGTAACAATGCCATGT
Gapdh	ACCCAGAAGACTGTGGATGG	GGATGCAGGGATGATGTTCT
Gdnf	CTTCGAGAAGCCTCTTACCG	GCCACTTGGAGTTAATGTCC
Fgf2	CATAGCAAGGTACCGGTTGG	CTCTACTGCAAGAACGGCG
Cxcl12	TGCATCAGTGACGGTAAACCA	TTCTTCAGCCGTGCAACAATC
Csf1	GGCTTGGCTTGGGATGATTCT	GAGGGTCTGGCAGGTACTC
Wt1	GAGAGCCAGCCTACCATCC	GGGTCCTCGTGTTTGAAGGAA
Gfar1	CACTCCTGGATTTGCTGATGT	AGTGTGCGGTACTTGGTGC
Zbtb16	CTGGGACTTTGTGCGATGTG	CGGTGGAAGAGGATCTCAAACA
Sohlh1	AGGAGGCGGATCTCGTTGA	GTCTGCAATCTGATTCGCCAG
Kit	TGTGGCTAAAGATGAACCCTC	ACACTCCAGAATCGTCAACTC
Sohlh2	GGGCAGGGCAGAGTAAATCTT	CAAACGAGTTAGCAGCCAAAAG
Dazl	ATACCTCCGGCTTATACAACTGT	GACTTCTTTTGCGGGCCATTT
Sycp1	CAAAAGCCCTTCACACTGTTCG	GTTTTCCCGACTGGACATTGTAA
Sycp3	AGCCAGTAACCAGAAAATTGAGC	CCACTGCTGCAACACATTCATA
Ddx4	GCTTCATCAGATATTGGCGAGT	GCTTGGAAAACCCTCTGCTT
Clgn	CCAGGGTGTTGGACTATGTTTG	CCCCGAGGAAGGTTCATCTTTA
tdTomato	CCTGTTCCTGGGGCATGG	TGATGACGGCCATGTTGTTG
Izumo1	ATGGGGCCGCATTTTACACTC	TCTTTAGCGCATCTGTCACAAA
Spem1	GGGTGGGCCTCGTATCAAAAC	ATGCGGATTTGGCTCCAGAG
Eif2c5	ATTGTGGTATGAATGTGAACT	GAAGCAGATGATAGATAATGGAA
Malat1	GTTACCAGCCCAAACCTCAA	CTACATTCCCACCCAGCACT

Table S4. Antibodies used for immunofluorescence

Antibodies description	SOURCE	IDENTIFIER
Anti-c-KIT Antibody	Abcam	ab283653
Anti-DDX4 Antibody	Abcam	ab270534/ab27591
Anti-SYCP3 Antibody	Santa Cruz	sc-74569
Anti-WT1 Antibody	Abcam	ab89901
Lectin PNA from Arachis hypogaea (peanut), Alexa Fluor® 488 conjugate	Abcam	ab70472
Goat anti-Mouse Alexa Fluor 488	Thermo Fisher Scientific	A-11001
Goat anti-Rabbit Alexa Fluor 488	Thermo Fisher Scientific	A-11008
Goat anti-Mouse Alexa Fluor 594	Thermo Fisher Scientific	A-11005
Goat anti-Rabbit Alexa Fluor 594	Thermo Fisher Scientific	A-11012
Goat anti-Rabbit Alexa Fluor 647	Thermo Fisher Scientific	A-21245
